# Obtaining patient phenotypes in SARS-CoV-2 pneumonia, and their association with clinical severity and mortality

**DOI:** 10.1186/s41479-024-00132-0

**Published:** 2024-06-25

**Authors:** Fernando García-García, Dae-Jin Lee, Mónica Nieves-Ermecheo, Olaia Bronte, Pedro Pablo España, José María Quintana, Rosario Menéndez, Antoni Torres, Luis Alberto Ruiz Iturriaga, Isabel Urrutia, COVID-19 & Air Pollution Working Group

**Affiliations:** 1 Basque Center for Applied Mathematics (BCAM), Bilbao, Basque Country Spain; 2 School of Science & Technology, IE University, Madrid, Madrid Spain; 3 Biocruces Bizkaia Health Research Institute, Barakaldo, Basque Country Spain; 4 Respiratory Service, Galdakao-Usansolo University Hospital, Galdakao, Basque Country Spain; 5 Research Unit, Galdakao-Usansolo University Hospital, Galdakao, Basque Country Spain; 6 Pneumology Department, La Fe University and Polytechnic Hospital, Valencia, Valencian Community Spain; 7 Pneumology Department, Hospital Clínic of Barcelona, Barcelona, Catalonia Spain; 8 Pneumology Service, Cruces University Hospital, Barakaldo, Basque Country Spain

**Keywords:** COVID-19, SARS-CoV-2 pneumonia, Phenotypes, Clustering, Unsupervised machine learning

## Abstract

**Background:**

There exists consistent empirical evidence in the literature pointing out ample heterogeneity in terms of the clinical evolution of patients with COVID-19. The identification of specific phenotypes underlying in the population might contribute towards a better understanding and characterization of the different courses of the disease. The aim of this study was to identify distinct clinical phenotypes among hospitalized patients with SARS-CoV-2 pneumonia using machine learning clustering, and to study their association with subsequent clinical outcomes as severity and mortality.

**Methods:**

Multicentric observational, prospective, longitudinal, cohort study conducted in four hospitals in Spain. We included adult patients admitted for in-hospital stay due to SARS-CoV-2 pneumonia. We collected a broad spectrum of variables to describe exhaustively each case: patient demographics, comorbidities, symptoms, physiological status, baseline examinations (blood analytics, arterial gas test), etc.

For the development and internal validation of the clustering/phenotype models, the dataset was split into training and test sets (50% each). We proposed a sequence of machine learning stages: feature scaling, missing data imputation, reduction of data dimensionality via Kernel Principal Component Analysis (KPCA), and clustering with the *k*-means algorithm. The optimal cluster model parameters –including *k*, the number of phenotypes– were chosen automatically, by maximizing the average Silhouette score across the training set.

**Results:**

We enrolled 1548 patients, each of them characterized by 92 clinical attributes (*d*=109 features after variable encoding). Our clustering algorithm identified *k*=3 distinct phenotypes and 18 strongly informative variables: Phenotype *A* (788 cases [50.9% prevalence] – age$\sim$57, Charlson comorbidity$\sim$1, pneumonia CURB-65 score$\sim$0 to 1, respiratory rate at admission$\sim$18 min^-1^, *Fi*O_2_$\sim$21%, C-reactive protein CRP$\sim$49.5 mg/dL [median within cluster]); phenotype *B* (620 cases [40.0%] – age$\sim$75, Charlson$\sim$5, CURB-65$\sim$1 to 2, respiration$\sim$20 min^-1^, *Fi*O_2_$\sim$21%, CRP$\sim$101.5 mg/dL); and phenotype *C* (140 cases [9.0%] – age$\sim$71, Charlson$\sim$4, CURB-65$\sim$0 to 2, respiration$\sim$30 min^-1^, *Fi*O_2_$\sim$38%, CRP$\sim$152.3 mg/dL).

Hypothesis testing provided solid statistical evidence supporting an interaction between phenotype and each clinical outcome: severity and mortality. By computing their corresponding odds ratios, a clear trend was found for higher frequencies of unfavourable evolution in phenotype *C* with respect to *B*, as well as more unfavourable in phenotype *B* than in *A*.

**Conclusion:**

A compound unsupervised clustering technique (including a fully-automated optimization of its internal parameters) revealed the existence of three distinct groups of patients – phenotypes. In turn, these showed strong associations with the clinical severity in the progression of pneumonia, and with mortality.

**Supplementary Information:**

The online version contains supplementary material available at 10.1186/s41479-024-00132-0.

## Introduction

Since the early stages of the worldwide COVID-19 pandemic outbreak, caused by the SARS-CoV-2 virus, a broad variety in the clinical evolution of patients was observed: from asymptomatic cases and mild affectations to critical cases and deadly respiratory failure. Such difference suggests the existence of distinct population groups who respond in notably disparate manners.

COVID-19 has fostered massive attention by the scientific community, who followed a wide spectrum of techniques and approaches: to improve our understanding about the behaviour of the disease, its transmission, diagnosis, therapy and prognosis, etc. Machine learning-based models provided predictions of severity and mortality which facilitated hospital resource allocation and aided in clinical decision making. In addition, several works in the literature have been devoted to discovering heterogeneous ‘phenotypes’ (i.e. clusters in the data science terminology) underlying in the population, and to associate them with eventual clinical outcomes: e.g. mortality, need for admission to intensive care units (ICU) or for mechanical ventilation, survival time and/or length of in-hospital stay.

This work aims to contribute towards the understanding of clinical phenotypes in COVID-19, obtained for a Spanish cohort of hospitalized patients with SARS-CoV-2 pneumonia; and to relate such phenotypes with two different clinical outcomes: severity in patients’ evolution and mortality.

### Related works

Wang et al. [1] examined *n*=20572 cases positive for COVID-19, of which 3548 required hospitalization. The study enrolled patients in the USA from March to October 2020 and incorporated data about patient’s demographics (age, sex), comorbidities and a selection of 17 biomarkers from routine blood tests. Using Latent Class Analysis (LCA) for clustering, the authors found 7 distinct phenotypes across the entire cohort, as well as 5 subphenotypes for the hospitalized population. Among these latter, the first subphenotype (14% prevalence) was formed by younger patients, with elevated counts of white blood cells (WBC) and platelets, mild anaemia and normal ranges of C-reactive protein (CRP), creatinine and albumin. The second subphenotype (21% prevalence) had mid-aged individuals with none or few comorbidities, lymphopenia and elevated CRP. The third (20%) had also mid-aged, but with more comorbidities, hyperinflammatory response and markedly high CRP, WBC and platelets. The fourth subphenotype (25%) were older patients, with the highest presence of comorbidity, leukopenia and lymphopenia. The fifth (20%) was also formed by old individuals, with a hyperinflammatory response and kidney dysfunction, high creatinine, anaemia, lymphopenia, hypoalbuminemia, elevated CRP, etc. In terms of clinical outcomes, 3 and 5 related to higher likelihoods of ICU admission and/or in-hospital death than 1 and 2; whereas 4 and 5 had more unfavourable survivals than the others – despite 3 going more often to ICU.

Su et al. [2] analyzed *n*=14418 patients from 5 hospitals based in the USA (16.3% treated in the emergency department, 83.7% hospitalized), for an enrolment period spanning between March and June 2020. The authors collected sociodemographic data (age, sex, race/ethnicity), 9 comorbidities and 30 biomarkers; selecting 23 variables after data quality assessment. Via hierarchical agglomerative clustering, they discover 4 underlying subphenotypes. Subphenotype I (33% prevalence) tended to include younger patients, more females and lower comorbidities. II (37%) had more males, more abnormal markers of inflammation (CRP, interleukin IL-6, lactate dehydrogenase LDH, erythrocyte, etc.) and hepatic dysfunction (ferritin, alanine, bilirubin). III (18%) encompassed older patients, with more frequent black ethnicity, renal dysfunction (blood urea nitrogen BUN, creatinine) and hematologic (D-dimer, hemoglobin). IV (12%) had also older patients, more males, higher comorbidity and more abnormal values across all biomarkers. The authors reported those subphenotypes to behave as a strong predictor for various clinical outcomes: most notably, for 60-day mortality. Interestingly, there also existed an association with the patient’s socioeconomic status. I had the most favourable outcomes (in terms of rates of death, need for mechanical ventilation and ICU admission), whereas II and III showed intermediate situations, and IV was the most unfavourable.

Lusczek et al. [3] enrolled *n*=1022 in-hospital patients from 14 centres in the USA, from March to August 2020. The authors collected 33 variables within the first 72 hours after admission: demographics (age, body mass index BMI), 9 comorbidity categories, vital signs (heart and respiratory rates, blood pressure, oxygenation *Sp*O_2_), and laboratory analyses. An ensemble consensus clustering –based on *k*-means– suggested the presence of 3 phenotypes, with statistically significant interactions with comorbidity, complications and hazard of death. Phenotype I (23% prevalence) was termed ‘adverse’: it included older patients, with more comorbidities (cardiac, hematologic, renal, although less resporatory), and altered LDH, neutrophils, D-dimer, aspartate aminotransferase AST, CRP, etc. It was associated with the most unfavourable clinical outcomes: in terms of mortality, mechanical ventilation and ICU. Phenotype II was the most common (60%) and represented an intermediate situation, with less hepatic disease than I or III but more comorbidity in general (e.g. metabolic and autoimmune). Phenotype III (17%) was ‘favourable’: with more females and more neutropenia, also more frequence smoking and/or alcohol abuse. Despite the very high rate of respiratory comorbidity, it showed the best clinical outcomes –lowest mortality–; and the authors hypothesized that they were more predisposed to long-term sequelae.

Besides, Gutiérrez et al. [4] conducted a clustering study with an internal cohort for phenotype derivation and internal validation (*n*=4035 patients from 127 hospitals in Spain, belonging to the first COVID-19 pandemic wave in the country, February to April 2020 – 66% of them for derivation, 34% for validation), alongside external validation (*n*=2226). Their dataset encompassed 69 variables per patient: age, sex, race/ethnicity, 16 comorbidities, 6 prior medication treatments, 7 COVID-19 symptoms, laboratory data and chest radiological findings. Through a two-step cluster analysis –in which the optimal number of clusters was found by maximizing the Silhouette score–, the authors identified 3 phenotypes. Phenotype *A* (19% prevalence) had younger individuals, less frequently males, with mild symptoms, normal inflammatory patterns (CRP, Il-6, ferritin, LDH) and higher lymphocytes. *B* (73%) showed cases with more symptoms (fever, cough), often without pulmonary infiltrations in chest X-ray but more interstitial, obesity, lymphocytopenia, and moderately elevated inflammatory parameters. Patients in *C* (7%) suffered more obesity, frequent comorbidities (hypertension, diabetes, chronic heart/lung/kidney diseases), poorer oxygenation, and even higher inflammatory biomarkers than *B* (neutrophils, D-dimer, procalcitonin, CRP). In turn, these phenotypes showed statistically remarkable differences in 30-day mortality rates: 3.7% for *A* in the external validation cohort, 23.7% for *B*, and 51.4% for *C*.

Ranard et al. [5] examined another USA cohort with *n*=528 hospitalized patients (March to July 2020), employing age and around 40 laboratory values (median and inter-quartile range throughout each patient’s hospitalization) as their input data. The authors trained a range of clustering algorithms, namely: *k*-means, Birch, Gaussian Mixture Models and agglomerative hierarchical; obtaining 4 phenotypes. Endotype 1 (25.6% prevalence) had the highest rate of women, the lowest hypertension and diabetes, but the highest chronic obstructive pulmonary disease; it encompasses the cases with the lowest inflammatory status (ferritin, IL-6, CRP, LDH), the lowest infectious status (WBC, procalcitonin), and the lowest coagulopathy (prothrombin time and partial thromboplastin time). Endotype 2 (18.9%) showed the most aggravated comorbidities (hypertension, diabetes, chronic kidney and renal diseases, heart failure), moderate inflammatory and infectious statuses, and low coagulopathy. Endotype 3 (32.0%) had low comorbidity, moderate inflammatory and infectious statuses, but high coagulopathy. Finally, endotype 4 (23.5%) had the fewest women, high comorbidity, high inflammatory and infectious statuses, and high coagulopathy. The authors reported evidence of statistical differences in mortality – increasing from 1 to 4; and in the ratio of intubations – below average for 1 and 2, above average for 3 and 4.

Teng et al. [6] considered *n*=483 hospitalized patients in the USA, enrolled between February and May 2020. The authors collected information on demographics (age, sex, race/ethnicity, BMI), 8 comorbidities, 8 laboratory variables and 8 types of medications during admission. With these, they found two phenotypes in their overall cohort via LCA. Cluster C1 (40% prevalence) encompassed older patients, fewer males, fewer individuals from non-white ethnicity, more comorbidities (hypertension, coronary, chronic heart failure, diabetes, kidney, pre-existing respiratory conditions, etc.), higher creatinine and pro-natriuretic peptide (pro-BNP), but lower inflammatory markers (CRP, alanine). Conversely, patients in cluster C2 (60%) were younger, more obese and with higher inflammatory markers (CRP, alanine). In terms of the observed clinical outcomes, these two clusters did not differ significantly in the length of stay, but they did for in-hospital death: 25.4% for C1 versus 9.0% for C2. Subsequently, the authors derived an extra clustering for the subpopulation of 75 deceased cases, although the resulting two subphenotypes (C1’, C2’) were statistically comparable to the overall ones (C1, C2).

Epsi et al. [7] investigated symptom clusters with *n*=1273 USA military patients from different pandemic waves, (March 2020 to March 2022), relating these symptoms to various clinical progressions (including failure to return to usual health and/or prolonged COVID-19). Methodologically, they exploited linear Principal Component Analysis (PCA) and *k*-means clustering – with the optimal *k* chosen by gap statistics. The authors reported three clusters: ‘Nasal’ (34% prevalence) –runny nose, sneezing– showcased intermediate comorbidity (40% cases with non-zero Charlson comorbidity index), and had a hospitalization rate (11.9%) lower than the overall average. ‘Sensory’ (35%) –loss of smell and/or taste– had individuals younger than in the other two clusters, with the lowest presence of comorbidity (28% non-zero Charlson), and also low hospitalization (10.5%). The ‘Respiratory/systemic’ cluster (31%) –upper and lower respiratory symptoms (cough, trouble breathing) and/or systemic (e.g. body ache)– entailed the worst comorbidity (47% non-zero Charlson), which translated to the highest hospitalization (36.3%) and other unfavourable outcomes: no-return to usual health and/or prolonged COVID-19 (beyond 6 months).

With a particular focus on the characterization of ICU patients, Chen et al. [8] recruited *n*=504 ICU cases in China, from January to March 2020. The authors collected 26 clinical variables: age, comorbidities, vital signs (heart and respiratory rates, blood pressure, oxygenation, etc.), and laboratory results within the first 24 h after ICU admission. Both consensus *k*-means clustering and LCA agreed on a two-phenotype model: the former determining *k* by gap statistics, the latter by minimization of the Akaike information criterion (AIC) for parsimoniousness. In addition, 5 out of the 26 variables –neutrophils vs. lymphocytes ratio NLR, *Sp*O_2_/*Fi*O_2_, LDH, tumour necrosis factor TNF-$\alpha$, and urea nitrogen) were selected attending to their informativeness –feature importance– as judged by various supervised machine learning classifiers of bagging and boosting types. The so-termed ‘hyperactive’ cluster (36% prevalence), when compared against the ‘hypoactive’ one (64%), encompassed: older patients, with more comorbidities, elevated heart and respiratory rates, higher Sequential Organ Failure Assessment (SOFA) score, elevated inflammation markers (e.g. WBC, NLR, CRP, IL-6, TNF-$\alpha$), and more extreme laboratory values regarding organ dysfunction (platelets, bilirubin, creatinine, urea nitrogen, LDH, *Sp*O_2_/*Fi*O_2_, etc.). Besides, these two clusters showed significant differences across all clinical outcomes of interest, not only 28-day mortality (74.3% for ‘hyperactive’ versus 10.8% for ‘hypoactive’) but also for frequency of acute respiratory distress, septic shock, acute cardiac and/or kidney injury and coagulopathy.

For Spain, Rodríguez et al. [9] studied a cohort formed by *n*=2022 ICU patients (February to May 2020). The authors investigated the association between phenotype and mortality risk. Having collected 42 clinical variables at ICU admission (age, sex, 13 comorbidities, APACHE II score for severity of illness, SOFA score for severity of organ dysfunction, 6 types of treatment and 8 laboratory measurements), they selected 25 of these variables as the most informative in relation to ICU mortality. By applying Partition Around Medoids (PAM) techniques, the authors found 3 phenotypes. Phenotypes *A* –‘mild’– and *B* –‘moderate’– showcased younger patients that *C* –‘severe’–; both with lower severity (APACHE II, SOFA), better inflammatory (LDH), renal (ferritin) and hematologic markers (D-dimer). Between *A* and *B*, the main differences are in D-dimer and in the presence of shock. Besides, their *C* cluster was reported to entail significant differences in clinical evolution with respect to the other two: particularly, higher ICU mortality (20.3% for *A*, 25.5% for *B*, and 45.4% for *C*).

In the Netherlands, Siepel et al. [10] collected data from *n*=2438 patients admitted to ICU, from February 2020 to March 2021 (the first and second COVID-19 pandemic waves in the country). They used 41 explanatory variables (demographics, clinical observations, medication, lab tests, vital signs and recordings of life support devices at the ICU) to describe the time-dependent evolution in the clinical status of patients. The authors conducted 21 day-by-day analyses. At admission and until ICU day 4, two clusters were reported to exist: ‘mild’ (38.2% prevalence) and ‘severe’ (61.8%). From then onwards, and until day 15, the ‘severe’ one split into ‘mild’ (38.2% prevalence) and ‘severe’ (36.3%). Throughout day 21, only 8.2% of the initial ‘mild’ cluster and only 4.6% of the initial ‘severe’ remained assigned to the same phenotype. This behaviour highlighted the suitability of time-dependent analyses. Besides, the authors pointed out that the heterogeneity appeared to be driven by inflammation biomarkers and dead space ventilation.

## Materials & methods

### Study design

Multicentric observational, prospective, longitudinal, cohort study conducted in four public hospitals from three geographical territories in Spain: Clínic Hospital (Barcelona, Catalonia), La Fe Hospital (Valencia, Valencian Community), as well as Galdakao-Usansolo and Cruces Hospitals (respectively located in Galdakao and Barakaldo, Basque Country). The study was approved by each local Ethics Committee for Clinical Research (corresponding reference codes: HCB/2020/0273, 20-122-1, PI 2019090, PI 2020083). It was carried out in adherence to the relevant guidelines and regulations: all participants provided voluntarily written informed consent before being enrolled in the study.

The inclusion criterion was adult patients ($\ge$ 18 years old) admitted to in-hospital stays due to SARS-CoV-2 pneumonia during the first epidemic wave of COVID-19 in Spain, between mid-February and the end of May 2020. Requirements for COVID-19 pneumonia diagnosis were:

i) a positive microbiological test (positive DNA amplification test by PCR for SARS-CoV-2); as well as
ii) compatible chest imaging findings (radiography and/or tomography).

Thus, patients hospitalized for SARS-CoV-2 infection without diagnosis of pneumonia, or who refused to participate or to sign the written informed consent, were excluded.

Two main clinical outcomes of interest were considered: mortality and severity in the evolution of SARS-CoV-2 pneumonia. For mortality, we accounted for those cases who either died during hospital stay or within 30 days after admission. For severity, we defined the following systematic objective criteria. High severity comprised patients who either:

- died intra-hospital or within 30 days after admission; or
- required major respiratory aids/aggressive treatments (high flow oxygen therapy, non-invasive mechanical ventilation, orotracheal intubation, extracorporeal membrane oxygenation, hemofilter, and/or vasoactives); or who
- were admitted to intensive care units (ICU) –including ‘intermediate’ respiratory ICUs–; or who
- suffered important clinical complications (e.g. distress, shock).

Medium severity was formed by cases who either:

- stayed in-hospital for at least 14 days, or
- suffered intermediate complications (e.g. pulmonary embolism, congestive heart failure, neurological deterioration, etc.).

Complementarily, the low severity group comprised the rest of the patients, whose clinical evolution was thus favourable.

A broad set of explanatory variables were collected per patient, including:

- demographics (e.g. age, sex, BMI, or whether the patient resides in a nursing home);
- pre-existing comorbidities;
- symptoms, physiological status and treatments prescribed during the preliminary emergency episode; and
- results from baseline examinations at the time of hospitalization (blood analytics, arterial gas tests, etc.).

### Data preparation

Data analyses were carried out *a posteriori*, after all individuals had been discharged.

As a first step, the dataset was pre-processed to guarantee its quality and integrity. We discarded any variables suffering from $\ge$60% missing data. Discrete categorical variables (e.g. type of bronchological comorbidity, type of pulmonary infiltration) were transformed into binary via one-hot encoding [11]; whereas discrete ordinal variables (e.g. smoking status: non-/ex-/smoker, or clinical pneumonia severity scores PSI and CURB-65) were treated as integer numeric data. Continuous variables spanning several orders of magnitude (e.g. most of the concentrations and cell counts in the blood tests) were $\log _{10}$-transformed.

### Phenotypes: clustering

First, we randomly partitioned our cohort into two disjoint subsets (50% data in each): for training and test purposes. In such division, we stratified with a double criterion (by hospital and by severity outcome), in order to guarantee an even distribution of cases.

**Fig. 1:**
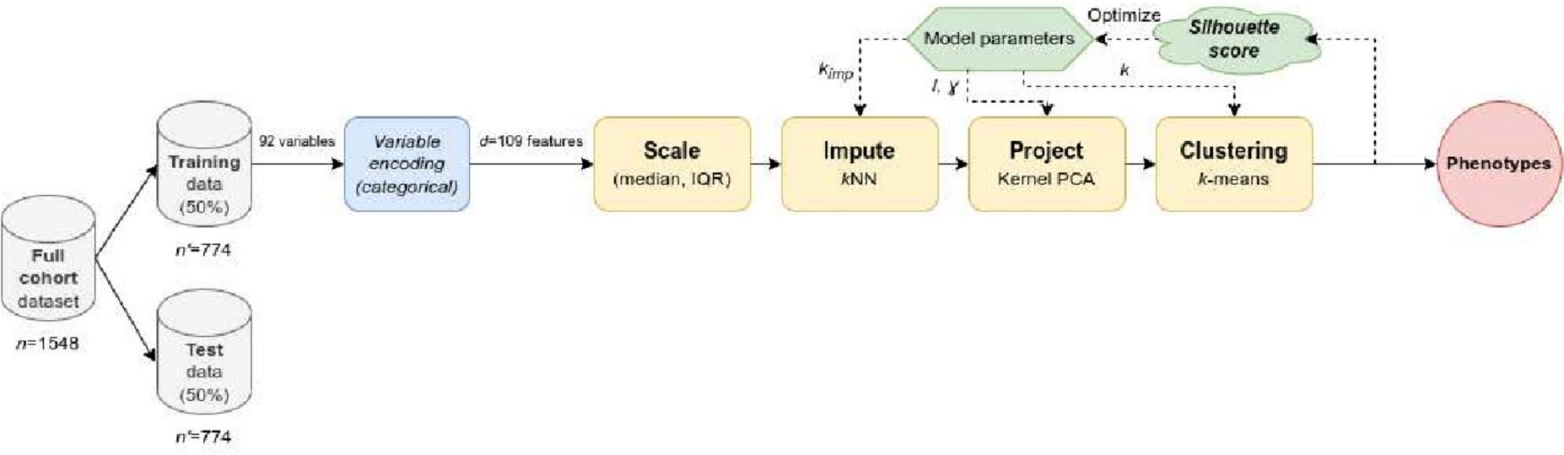
Steps to obtain patient phenotypes via clustering techniques

To obtain suitable phenotypes, unsupervised clustering algorithms were employed. Specifically, we proposed a sequence with four stages (Fig. 1):

1. Scaling – For the sake of robustness against outliers, we scaled our data based on each variable’s median and inter-quartile range; instead of the classical normalization by mean and standard deviation.
2. Imputation of missing values – Via *k*-nearest neighbours techniques (*k*NN) [12].
3. Projection (i.e. reduction of data dimensionality) – We retained the most relevant information by means of the Kernel Principal Component Analysis (KPCA) algorithm [13], which is a non-linear generalization of the classical PCA through the use of kernels. In particular, we opted for radial basis function (RBF) kernels.
4. Unsupervised clustering – Using *k*-means clustering.

To prevent information leakage [14], not only the final clustering stage was fitted on the training set; but instead the whole sequence of techniques (Fig. 1): scaling, imputation, projection and clustering. In this process, a suitable combination of four key parameters must be selected, to control the internal behaviour of the algorithms. These parameters are:

i) The number $k_{imp}$ of neighbours for *k*NN imputation (Options explored: 5 or 9 neighbours).
ii) The number *l* of components projected by KPCA (Options explored: 2, 3, 5 or 10 KPCA components).
iii) KPCA’s RBF kernel coefficient $\gamma$ (Range explored: from $10^{-4}$ to 0.02 in geometric progression, along with the default $\gamma =^{1}\!\!\!/_{\!d}$, where *d* is the number of features prior to projection).
iv) The final number *k* of clusters –i.e. phenotypes– for *k*-means (Options explored: 3 to 8 clusters).

We proceeded by maximizing the average Silhouette score [15], as a measure of cluster consistency, over the training dataset. Subsequently, the learnt model was applied on the test cohort, to ascertain its validity and reproducibility.

Our analysis was implemented in Python programming language, via the software library for machine learning scikit-learn [16].

### Phenotypes: *post-hoc* statistical analyses

We did not conduct any *a priori* statistical sample size calculation. Instead, the size of our cohort was equal to the number of SARS-CoV-2 pneumonia patients fulfilling the inclusion criteria during the pre-established period of enrollment.

First, we studied the interaction relationships among:

- phenotype,
- clinical outcome –either severity (ternary: [low-medium-high]) or mortality (binary: [survived-deceased])–, and
- training/test data partitions.

Hence, we examined three-way contingency tables for mutual (i.e. complete) and joint independence (phenotype and clinical outcome, against train/test) by means of $\chi ^2$ tests [17].

Subsequently, we calculated the corresponding odds ratios (OR) and 95% confidence intervals (CI) for both clinical outcomes, by phenotype and by data partition (i.e. full cohort on the one hand, training/test sets on the other). To do so, we used Fisher’s exact test [18].

In addition, for each of the demographic and clinical variables collected in this study, we carried out univariate analyses to ascertain the statistical difference of values across phenotypes, as well as their corresponding effect size (i.e. the statistical magnitude of strength for such differences) [19]. For discrete variables, we employed univariate $\chi ^2$ tests and bias-corrected Cramer’s *V* for effect size [20]. For continuous variables, we used the non-parametric Kruskal-Wallis test and its corresponding $\eta _H^2$ effect size. Thresholds for effect size interpretation were taken from [21].

These statistical analyses were carried out with the Python library for scientific computing SciPy [22] and with the statistical software JASP [23].

## Results

### Cohort

Attending to our inclusion and exclusion criteria, a total of *n*=1548 patients were enrolled in this study. From the demographic and clinical information collected at the baseline time of hospitalization, 92 explanatory variables met our criterion of <60% missingness: whereas other 14 variables –e.g. ferritin, bilirubin, albumin, troponin, interleukin-6 (IL-6), aspartate aminotransferase (AST), creatine phosphokinase (CPK), platelets or eosinophils– failed to match this data quality criterion (see [24] for further details). Once categorical variables were transformed via one-hot encoding, these 92 attributes became *d*=109 features.

A comprehensive description of the characteristics of our cohort can be found in the on-line supplementary materials (Appendix A: Table A.1, Figs. A.1 to A.7). However, patient confidentiality issues prevented us from making the full dataset publicly available.

### Phenotype extraction

Following the methodology detailed in “Phenotypes: clustering” section and Fig. 1, our automated selection for optimal clustering parameters resulted in *k*NN imputation with $k_{imp}$=9 neighbours, KPCA projection onto *l*=2 dimensions with its RBF kernel coefficient $\gamma =^{1}\!\!\!/_{\!d}\approx 9.174 \cdot 10^{-3}$, and *k*=3 clusters – onwards denoted phenotypes *A*, *B* and *C*. This process yielded an average Silhouette score of 0.4914 for the training set, along with a Silhouette score of 0.4775 once the fitted model was applied to the test set.

**Fig. 2:**
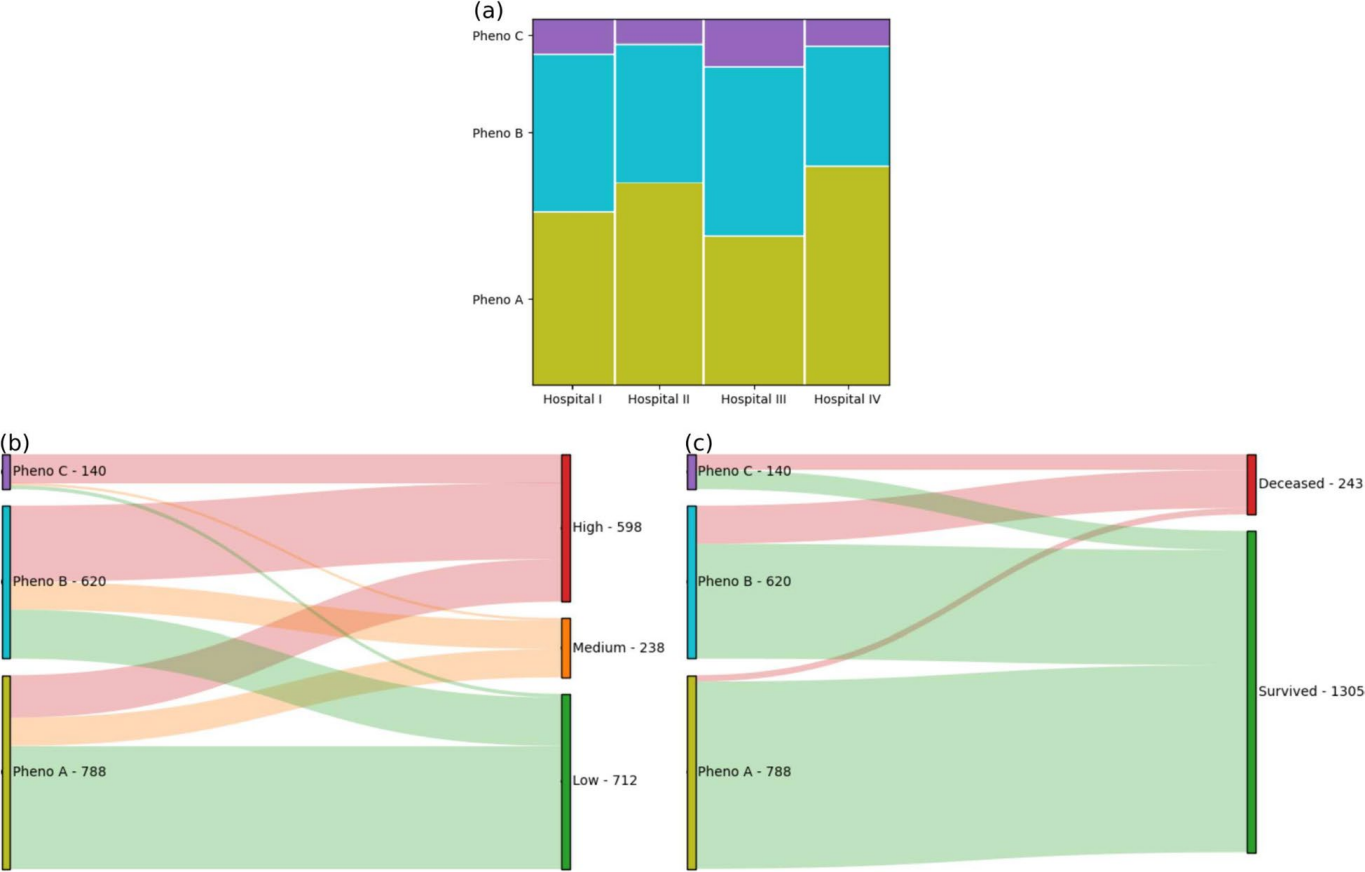
Distribution of patients: by phenotype, hospital and clinical outcome (severity, mortality) – **a** Cases by hospital and phenotype. **b**, **c** Sankey flow diagrams for the relationship between phenotype [left] and outcome [right]: **b** severity, or **c** mortality

Table 1 and Fig. 2 reflect the distribution of patients, by hospital (anonymized into I-IV) as well as by clinical outcome, along with the number of correspondences obtained for phenotypes *A*, *B* and *C*. Note that our double stratification was able to successfully guarantee a balanced distribution of cases –by hospital and by severity– between the training and test sets.

When focusing on SARS-CoV-2 pneumonia severity as the clinical outcome of interest, the $\chi ^2$ test for mutual independence [*Phenotype*, *Severity*, *Training/Test Partition*] resulted strongly significant ($p\ll$0.001), hence rejecting the null hypothesis of complete independence. In addition, the test for joint independence [*Phenotype & Severity*, *Training/Test*] was non-significant (*p*=0.6530). Complementarily, with mortality as outcome, the $\chi ^2$ test for mutual independence [*Phenotype*, *Mortality*, *Training/Test*] was also strongly significant ($p\ll$0.001); whereas the joint independence [*Phenotype & Mortality*, *Training/Test*] was again non-significant (*p*=0.3696). Therefore, we can conclude that there exists solid statistical evidence supporting an interaction between phenotype and both clinical outcomes, regardless of the data partition (training/test). Consequently, we will onwards analyze further results aggregating both partitions into the full cohort data, unless explicitly stated otherwise.

**Table 1:** Number of patients, by data partition and phenotype

		Full cohort				Training set				Test set			
		*Total*	*By phenotype*			*Total*	*By phenotype*			*Total*	*By phenotype*		
			*A*	*B*	*C*		*A*	*B*	*C*		*A*	*B*	*C*
***Hospital***	*I*	358 (23.1%)	171	154	33	179	74	91	14	179	97	63	19
	*II*	380 (24.5%)	212	143	25	190	103	74	13	190	109	69	12
	*III*	438 (28.3%)	180	202	56	219	87	104	28	219	93	98	28
	*IV*	372 (24.0%)	225	121	26	186	113	59	14	186	112	62	12
***Severity***	*Low*	712 (46.0%)	500	197	15	355	240	109	6	357	260	88	9
	*Medium*	238 (15.4%)	115	113	10	120	54	62	4	118	61	51	6
	*High*	598 (38.6%)	173	310	115	299	83	157	59	299	90	153	56
***Mortality***	*Survived*	1305 (84.3%)	762	465	78	655	366	252	37	650	396	213	41
	*Deceased*	243 (15.7%)	26	155	62	119	11	76	32	124	15	79	30
***Overall***		1548	788	620	140	774	377	328	69	774	411	292	71
			(50.9%)	(40.0%)	(9.0%)		(48.7%)	(42.4%)	(8.9%)		(53.1%)	(37.7%)	(9.2%)

Table 2 summarizes the ORs for each clinical outcome, disaggregated by phenotypes as well as by data partition: either full cohort or training/test. Table 2 outlines a clear trend –also visible in Fig. 2b, c– where phenotype *A* (788 patients, 50.90%) is consistently and significantly related to lower SARS-CoV-2 pneumonia severity ($p<$0.001), and to a decreased risk of death ($p<$0.001). On the contrary, phenotype *B* (620 patients, 40.05%) –and much more markedly phenotype *C* (140 patients, 9.04%)– are associated with increasingly unfavourable outcomes: higher odds for medium ($p<$0.05 in most cases) and high ($p<$0.001) severity cases, and more prevalent mortality ($p<$0.001).

Furthermore, Table 2 is congruent with our previous results regarding joint independence [*Phenotype*, *Clinical outcome*, *Partition*], such as the ORs for training and test are always comparable: with each other, and against the OR for the full cohort.

**Table 2:** Odds ratios (OR) for clinical outcomes, by phenotype – Mean [95% CI]

		Phenotype A			Phenotype B			Phenotype C		
		*Cohort*	*Training*	*Test*	*Cohort*	*Training*	*Test*	*Cohort*	*Training*	*Test*
***Severity***	*Low*	4.4829	4.2870	4.7114	0.3737	0.4051	0.3421	0.1225	0.0974	0.1484
		[3.5994, 5.5959]	[3.1414, 5.8774]	[3.4344, 6.4979]	[0.3000, 0.4646]	[0.2975, 0.5498]	[0.2478, 0.4698]	[0.0659, 0.2126]	[0.0340, 0.2280]	[0.0638, 0.3063]
		$p<$0.001	$p<$0.001	$p<$0.001	$p<$0.001	$p<$0.001	$p<$0.001	$p<$0.001	$p<$0.001	$p<$0.001
	*Medium*	0.8850	0.8386	0.9357	1.4314	1.5583	1.3103	0.3983	0.3128	0.4875
		[0.6646, 1.1780]	[0.5553, 1.2624]	[0.6200, 1.4137]	[1.0733, 1.9077]	[1.0347, 2.3500]	[0.8612, 1.9852]	[0.1837, 0.7718]	[0.0812, 0.8652]	[0.1685, 1.1579]
		*p*=0.3983	*p*=0.4269	*p*=0.7643	*p*=0.0119	*p*=0.0272	*p*=0.1818	*p*=0.0031	*p*=0.0218	*p*=0.1178
	*High*	0.2220	0.2370	0.2071	2.2210	1.9638	2.5298	8.7970	11.3933	7.0488
		[0.1766, 0.2783]	[0.1705, 0.3274]	[0.1491, 0.2858]	[1.7909, 2.7567]	[1.4484, 2.6668]	[1.8535, 3.4607]	[5.5796, 14.3584]	[5.6520, 25.4517]	[3.8353, 13.7154]
		$p<$0.001	$p<$0.001	$p<$0.001	$p<$0.001	$p<$0.001	$p<$0.001	$p<$0.001	$p<$0.001	$p<$0.001
***Mortality***		0.0855	0.0806	0.0885	3.1793	2.8226	3.5956	5.3797	6.1221	4.7268
		[0.0538, 0.1310]	[0.0384, 0.1538]	[0.0468, 0.1568]	[2.3714, 4.2823]	[1.8511, 4.3479]	[2.3698, 5.5065]	[3.6546, 7.9024]	[3.4975, 10.6876]	[2.7092, 8.1871]
		$p<$0.001	$p<$0.001	$p<$0.001	$p<$0.001	$p<$0.001	$p<$0.001	$p<$0.001	$p<$0.001	$p<$0.001

### Phenotype description

**Fig. 3:**
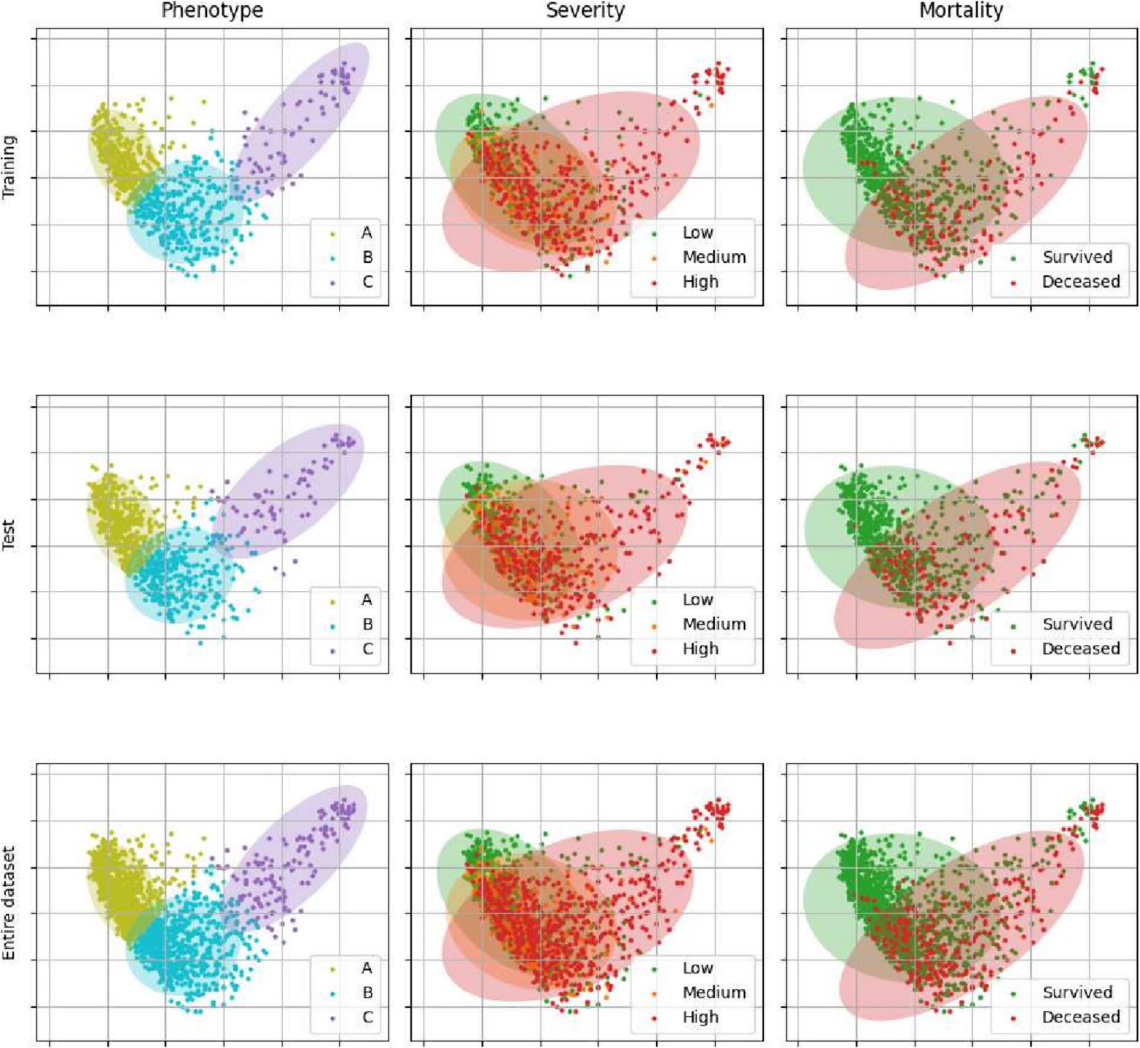
Two-dimensional projection of our data via Kernel Principal Component Analysis (KPCA): Training set (upper row), test set (middle row) and full cohort (bottom row). Points represent patients, whereas the shadowed areas depict the corresponding 95% confidence ellipses: For clusters/phenotypes *A–C* (left column); for low-medium-high severity as clinical outcome (central column); and for mortality (right column)

Figure 3 depicts two-dimensional projections of our data by means of KPCA (*l*=2), including the associated 95% confidence ellipses. Clusters are prominently separable, whereas clinical outcomes entail an important degree of overlapping – most notably, medium severity (orange). Again, these plots visually reaffirm the validity and reproducibility of the phenotypes derived from the training set, when applied to the test set.

**Table 3:** Main characteristics of our cohort, overall and by phenotype – Descriptive statistics of those 18 variables selected for having a large effect size across the *k*=3 phenotypes

*Variable*		Overall	By phenotype					
			*Pheno A*	*Pheno B*	*Pheno C*			
		*n*=1548	*n*=788 (50.9%)	*n*=620 (40.0%)	*n*=140 (9.0%)	*p*-value	Effect size	
*Age* $\left[ years\right]$	Median	65	57$^{\mathbb{B}\mathbbm{c}}$	75$^{\mathbb{A}\mathbbm{c}}$	71$^{\mathbb{a}\mathbbm{b}}$	K-W	$\eta _{H}^{2}$	
	IQR	[53, 77]	[46, 66]	[66, 83]	[59, 82]			
	Num. valid	1548 (100%)	788 (100%)	620 (100%)	140 (100%)	<0.001	0.284	Large
*Comorbidity: Charlson index*	Median	3	1$^{\mathbb{B}\mathbbm{c}}$	5$^{\mathbb{A}\mathbbm{c}}$	4$^{\mathbbm{a}\mathbbm{b}}$	K-W	$\eta _{H}^{2}$	
	IQR	[1, 5]	[0, 3]	[3, 7]	[2, 6]			
	Num. valid	1548 (100%)	788 (100%)	620 (100%)	140 (100%)	<0.001	0.351	Large
*Pneumonia: PSI score*	Median	70	55$^{\mathbb{B}\mathbbm{c}}$	91$^{\mathbb{A}}$	95$^{\mathbbm{a}}$	K-W	$\eta _{H}^{2}$	
	IQR	[53, 92]	[45, 68]	[76, 114]	[76, 114]			
	Num. valid	1287 (83.1%)	705 (89.5%)	479 (77.3%)	103 (73.6%)	<0.001	0.433	Large
*Pneumonia: CURB-65 score*	0	539 (34.8%)	468 (59.4%)$^{\mathbb{B}\mathbb{C}}$	49 (7.9%)$^{\mathbb{A}\mathbbm{c}}$	22 (15.7%)$^{\mathbb{A}\mathbbm{b}}$	$\chi ^{2}$	*V*	
	1	516 (33.3%)	257 (32.6%)	218 (35.2%)	41 (29.3%)			
	2	361 (23.3%)	50 (6.3%)	261 (42.1%)	50 (35.7%)			
	3	89 (5.7%)	4 (0.5%)	66 (10.6%)	19 (13.6%)			
	4	20 (1.3%)	0	12 (1.9%)	8 (5.7%)			
	NA	23 (1.5%)	9 (1.1%)	14 (2.3%)	0	<0.001	0.439	Large
*Admission status: Respiratory rate *$\left[ min^{-1}\right]$	Median	18	18$^{\mathbbm{b}\mathbbm{c}}$	20$^{\mathbbm{a}\mathbbm{c}}$	30$^{\mathbbm{a}\mathbbm{b}}$	K-W	$\eta _{H}^{2}$	
	IQR	[16, 24]	[16, 20]	[17, 25]	[23, 32]			
	Num. valid	1021 (66.0%)	577 (73.2%)	364 (58.7%)	80 (57.1%)	<0.001	0.149	Large
*Admission status: *$FiO_{2}$ $\left[ fraction\right]$	Median	0.21	0.21$^{\mathbbm{b}\mathbb{C}}$	0.21$^{\mathbbm{a}\mathbb{C}}$	0.38$^{\mathbb{A}\mathbb{B}}$	K-W	$\eta _{H}^{2}$	
	IQR	[0.21, 0.21]	[0.21, 0.21]	[0.21, 0.21]	[0.21, 0.83]			
	Num. valid	1526 (98.6%)	782 (99.2%)	608 (98.0%)	136 (97.1%)	<0.001	0.420	Large
*Admission status: *$SpO_{2}/FiO_{2}$ $\left[ ratio\right]$	Median	452.38	457.14$^{\mathbbm{b}\mathbb{C}}$	447.62$^{\mathbbm{a}\mathbbm{c}}$	238.10$^{\mathbb{A}\mathbbm{b}}$	K-W	$\eta _{H}^{2}$	
	IQR	[433.33, 461.90]	[447.62, 461.90]	[423.81, 457.14]	[105.63, 317.86]			
	Num. valid	1520 (98.2%)	781 (99.1%)	604 (97.4%)	135 (96.4%)	<0.001	0.300	Large
*Admission status: *$SpO_{2}/RespRate$ $\left[ \%/min^{-1}\right]$	Median	5.17	5.44$^{\mathbbm{b}\mathbbm{c}}$	4.70$^{\mathbbm{a}\mathbbm{c}}$	2.97$^{\mathbbm{a}\mathbbm{b}}$	K-W	$\eta _{H}^{2}$	
	IQR	[4.00, 5.94]	[4.75, 6.00]	[3.65, 5.56]	[2.29, 3.92]			
	Num. valid	1017 (65.7%)	577 (73.2%)	363 (58.5%)	77 (55.0%)	<0.001	0.178	Large
*Blood test: Urea* $\left[ mg/dL\right]$	Median	34	28$^{\mathbb{B}\mathbbm{c}}$	48$^\mathbb {A}$	48$^{\mathbbm{a}}$	K-W	$\eta _{H}^{2}$	
	IQR	[26, 47]	[22, 33]	[38, 64]	[35, 78]			
	Num. valid	1107 (71.5%)	606 (76.9%)	418 (67.4%)	83 (59.3%)	<0.001	0.411	Large
*Blood test: Creatinine* $\left[ mg/dL\right]$	Median	0.92	0.82$^{\mathbbm{b}\mathbbm{c}}$	1.09$^{\mathbbm{a}}$	1.04$^{\mathbbm{a}}$	K-W	$\eta _{H}^{2}$	
	IQR	[0.75, 1.13]	[0.69, 0.96]	[0.88, 1.50]	[0.84, 1.44]			
	Num. valid	1476 (95.3%)	763 (96.8%)	585 (94.4%)	128 (91.4%)	<0.001	0.213	Large
*Blood test: Blood urea nitrogen (BUN)* $\left[ mg/dL\right]$	Median	17.0	15.0$^{\mathbb{B}\mathbbm{c}}$	20.3$^{\mathbb{A}}$	20.0$^{\mathbbm{a}}$	K-W	$\eta _{H}^{2}$	
	IQR	[13.0, 23.0]	[12.0, 20.0]	[15.0, 27.0]	[15.0, 28.5]			
	Num. valid	736 (47.5%)	382 (48.5%)	297 (47.9%)	57 (40.7%)	<0.001	0.390	Large
*Blood test: C-reactive protein (CRP)* $\left[ mg/L\right]$	Median	72.13	49.51$^{\mathbbm{b}\mathbbm{c}}$	101.63$^{\mathbbm{a}\mathbbm{c}}$	152.60$^{\mathbbm{a}\mathbbm{b}}$	K-W	$\eta _{H}^{2}$	
	IQR	[32.30, 134.04]	[22.99, 91.90]	[50.09, 162.21]	[96.82, 256.47]			
	Num. valid	1473 (95.2%)	759 (96.3%)	586 (94.5%)	128 (91.4%)	<0.001	0.167	Large
*Blood test: Procalcitonin (PCT)* $\left[ \mu g/L\right]$	Median	0.11	0.07$^{\mathbb{B}\mathbbm{c}}$	0.18$^{\mathbb{A}}$	0.25$^{\mathbbm{a}}$	K-W	$\eta _{H}^{2}$	
	IQR	[0.06, 0.22]	[0.04, 0.12]	[0.10, 0.44]	[0.12, 0.81]			
	Num. valid	1089 (70.3%)	549 (69.7%)	440 (71.0%)	100 (71.4%)	<0.001	0.285	Large
*Blood test: Neutrophils vs. lympho* $\left[ ratio\right]$	Median	4.98	3.83$^{\mathbbm{b}\mathbbm{c}}$	7.13$^{\mathbbm{a}}$	9.68$^{\mathbbm{a}}$	K-W	$\eta _{H}^{2}$	
	IQR	[3.33, 8.61]	[2.72, 5.37]	[4.43, 11.75]	[5.57, 14.85]			
	Num. valid	1529 (98.8%)	781 (99.1%)	610 (98.4%)	138 (98.6%)	<0.001	0.218	Large
*Blood test: D-dimer* $\left[ ng/mL\right]$	Median	751	540$^{\mathbbm{b}\mathbbm{c}}$	1079$^{\mathbbm{a}}$	1286$^{\mathbbm{a}}$	K-W	$\eta _{H}^{2}$	
	IQR	[430, 1340]	[350, 865]	[636, 2100]	[819, 2406]			
	Num. valid	1268 (81.9%)	679 (86.2%)	482 (77.7%)	107 (76.4%)	<0.001	0.185	Large
*Blood test: Prothrombin index* $\left[ \%\right]$	Median	93	99$^{\mathbbm{b}\mathbbm{c}}$	84$^{\mathbbm{a}}$	91$^{\mathbbm{a}}$	K-W	$\eta _{H}^{2}$	
	IQR	[81, 100]	[89, 100]	[62, 96]	[81, 100]			
	Num. valid	662 (42.8%)	346 (43.9%)	264 (42.6%)	52 (37.1%)	<0.001	0.171	Large
*Arterial blood gas test:* $FiO_{2}$ $\left[ fraction\right]$	Median	0.21	0.21$^{\mathbbm{b}\mathbb{C}}$	0.21$^{\mathbbm{a}\mathbb{C}}$	0.21$^{\mathbb{A}\mathbb{B}}$	K-W	$\eta _{H}^{2}$	
	IQR	[0.21, 0.21]	[0.21, 0.21]	[0.21, 0.21]	[0.21, 0.80]			
	Num. valid	946 (61.1%)	474 (60.2%)	381 (61.5%)	91 (65.0%)	<0.001	0.199	Large
*Arterial blood gas test:* $SatO_{2}/FiO_{2}$ $\left[ ratio\right]$	Median	452.38	457.14$^{\mathbbm{b}\mathbb{C}}$	442.86$^{\mathbbm{a}\mathbbm{c}}$	270.00$^{\mathbb{A}\mathbbm{b}}$	K-W	$\eta _{H}^{2}$	
	IQR	[420.24, 461.90]	[447.62, 461.90]	[400.00, 457.14]	[124.64, 395.68]			
	Num. valid	730 (47.2%)	374 (47.5%)	294 (47.4%)	62 (44.3%)	<0.001	0.220	Large

*IQR* Inter-quartile range, *NA* Not available, *K-W* Kruskal-Wallis test, *NS*: not significant, *PSI* Pneumonia severity index, *CURB-65* Pneumonia severity score (confusion, urea, respiratory rate, blood pressure, age 65)

Pairwise *post hoc* comparisons – $\mathbb {P}$: significant difference with respect to phenotype *P*, large effect size; $\mathbbm{p}$: significant wrt *P*, medium or small effect size. Discrete variables with *post hoc* $\chi ^{2}$ and Cramer’s *V*; continuous with Dunn test and *r* effect size

As explained in “Phenotypes: post-hoc statistical analyses” section and in order to characterize the clusters identified here, we conducted univariate analyses to determine which of the 92 demographic and clinical attributes were significantly different across the *k*=3 phenotypes. Table A.1 [On-line supplementary materials] contains a full description of such results, including comparative graphs of distributions per phenotype. Table 3 summarizes the 18 variables (out of those 92 available) which were not only significant, but which furthermore showed a large statistical effect size [19]: hence implying that a prominent magnitude of inter-phenotype differences was found. In other words, Table 3 entails a compact clinical characterization of each of the three phenotypes *A*, *B*, *C* found. In the rightmost columns, pairwise *post hoc* comparisons of phenotypes are shown. As above, for the discrete variable we employed *post hoc* univariate $\chi ^2$ tests with Bonferroni correction (for multiple comparisons), and Cramer’s *V* effect size. In turn, for continuous variables we used the non-parametric *post hoc* Dunn test with Bonferroni correction, and its corresponding *r* effect size [25]. Thresholds for effect size interpretation were taken from [21].

## Discussion

This work is a clustering study, aimed at identifying clinical phenotypes across in-hospital COVID-19 patients from clinical data, routinely available early after admission. Our study comprised a cohort with *n*=1548 in-hospital patients with SARS-CoV-2 pneumonia, from four hospitals in three heterogeneous geographical areas of Spain. Compared to other works in the literature, which tackled the task of deriving clinical phenotypes for COVID-19, this cohort is of an intermediate size. Nevertheless, the range of demographic and clinical information collected here (92 explanatory variables, corresponding to *d*=109 features after encoding) is notably wider. Thus, this dataset allowed us to work on a comprehensive and exhaustive characterization of our cohort across different clinical domains: from patients’ demographics (age, sex, BMI, etc.) to pre-existing comorbidities, as well as general and COVID-19-specific symptoms at the time of admission, baseline physiological status and vital signs (e.g. pneumonia’s PSI and CURB-65 scores, body temperature, respiratory rate, oxygen saturation *Sp*O_2_ and inspired fraction *Fi*O_2_, etc.), treatments prescribed during the preceding emergency episode, baseline blood analytics at admission (urea, creatinine, CRP, procalcitonin, LDH, etc.) and arterial gas tests, among others.

Our cohort encompassed noticeable heterogeneity in the presentation of the clinical manifestations of SARS-CoV-2, as well as a remarkable diversity in the management of severe COVID-19 patients [26]. This heterogeneity further motivates the identification of patient subgroups, or phenotypes with similar clinical characteristics [1–10].

Using unsupervised machine learning approaches, we identified three phenotypes based on demographics, pre-existing conditions, clinical status (e.g. oxygenation) at presentation and laboratory data (biomarkers), across hospital admissions with SARS-CoV-2 pneumonia. These phenotypes yielded consistent and statistically significant associations with different odds ratios of mortality and severity. Therefore, the phenotypes could indicate different underlying mechanisms of the disease. Furthermore, the identification of high-risk profiles may enhance the procedures for patient inclusion in clinical trials specifically investigating suitable therapies for such high-risk profiles.

Indeed, patient phenotyping may play a key role in expanding our understanding of disease heterogeneity [27], and for higher success rates in treatment. Bruse et al. [28] employed four phenotypes extracted from non-COVID sepsis patients, and applied them successfully to critical COVID-19 sepsis patients. The fact that these authors identified comparable responses for each phenotype across the two sepsis subpopulations –COVID-19 or not– underlines the overall suitability and biological plausibility of approaches based on patient phenotyping.

We performed the training of our compound clustering model through a fully automated and reproducible selection of its key internal parameters, via an optimization of the average Silhouette score. Various types of findings confirmed the model’s satisfactory validity properties and generalization capabilities. Namely: joint independence tests [*Phenotype* & *Clinical outcome*] versus [*Partition*] (“Phenotype extraction” section), comparable ORs for training/test (Table 2), and visual inspections of KPCA’s 2D projection plots (Fig. 3).

For the sake of clinical interpretability, out of the three phenotypes found, *A* could be termed ‘protective’, since it was significantly associated with higher odds of low severity progression and with reduced mortality risks ($p<$0.001). Conversely, phenotype *B* could be interpreted as moderately ‘endangering’; whereas *C* as markedly ‘adverse’, in view of its association with increased odds of high severity and mortality ($p<$0.001). In particular, phenotype *A* showed in overall the most favourable evolutions: these patients could have been managed in a conventional ward, or –in the absence of respiratory failure– even be referred for out-of-hospital care. As for phenotype *B*, patients could have been placed in the ward and undergone periodic evolutionary controls.

In this regard, phenotype delineation may enhance the profiling of patients for precision medicine, helping to guide the prognosis in the evolution of the disease. Thus, phenotyping can play a positive role in medical resource allocation and hospital capacity planning, as well as in exploring more specific therapeutic strategies and evidence-based subgrouping in clinical trials. The phenotypes identified may allow the detection of that subgroup of patients with worse prognosis versus those with better prognosis. Hence, they can be used to institute the most appropriate treatment measures for each case, towards precision medicine.

Table 3 covers the main clinical characterization of our clustering results per explanatory variable; focused on those $^{18}\!/_{\!92}$ variables with statistically large inter-phenotype effect sizes. This subset of 18 key variables can also be viewed as a data-driven selection of the most informative factors for predicting the clinical outcomes under study (severity, mortality) [24, 29]. In particular, phenotype *C* (low prevalence, 9.0%) included older patients, with more comorbidities, worse respiratory status (peripheral oxygenation, as well as in the arterial blood gas tests), and more unfavourable inflammatory, renal and/or hematologic biomarkers (C-reactive protein, procalcitonin, D-dimer, neutrophils-to-lymphocyte ratio, creatinine, BUN, prothrombin, etc.) – Table 3. These findings are in line with independent literature, which pointed out such factors as prognostics of unfavourable evolution [30, 31], and potential targets for more aggressive therapy [32, 33].

Phenotype *B* (40.0% prevalence) showcases an intermediate situation between *A* and *C*. Whereas in terms of the respiratory status *B* is closer to *A* (i.e. milder cases), in comorbidity (Charlson index) and in biomarkers of renal function (urea, creatinine, BUN), *B* is more similar to *C*. Biomarkers of inflammation (mainly CRP, but also to a lesser extent procalcitonin, NLR – Table 3) differ substantially across phenotypes. *A* is the most prevalent (50.9%) and is associated with notably lower mortality odds and milder severity – Table 2.

Compared to related works in the literature (“Related works” section), our results here are consistent to an important extent. In [1], various of the main variables which outlined phenotypes apart are common with our findings: age, comorbidity, inflammatory and renal response (CRP, creatinine). Others (e.g. platelets, albumin) were not available for us to use due to data missingness (“Cohort” section). In [2], again: age, comorbidity, inflammation (CRP), renal (BUN, creatinine) and hematologic biomarkers (D-dimer) were decisive. Other factors (ferritin, alanine, IL-6) were scarcely represented in our data collection – due to the unprecedented clinical load in the pandemic scenario. In [3], findings about comorbidity, CRP, neutrophils and D-dimer were reported. Unlike in our work, LDH was decisive for them. In [4], COVID-19 symptoms changed significantly from one cluster to another, a behaviour that we did not observe. Nonetheless, inflammation (neutrophils, D-dimer, procalcitonin, CRP) and oxygenation distinguished the clusters, thus in agreement with our work. In [5], the thematic variables were comorbidity, inflammation, infectious status and coagulopathy (including prothrombin time). Sex diferences were manifest, unlike here. In [6], sex was again differential, along with comorbidity and inflammation (CRP, creatinine, alanine ALT).

The work by Epsi et al. [7] is arguably more difficult to compare with ours, since the former focused on demographics and symptoms only; yet comorbidity was decisive. On the other hand, the cohorts in [8–10] comprise ICU patients, meaning that the clinical scenarios are unmatched. Yet [8] highlighted the importance of comorbidity, inflammation (CRP, NLR) and biomarkers of organ dysfunction (LDH, BUN, platelets, bilirubin, etc.). In [9] age, LDH, D-dimer and ferritin are among the key factors; whereas [10] stressed again the role of inflammation and ventilation.

Not surprisingly and in good agreement with the comparable literature –see the narration above, and in “Related works” section–, patients with a disfavourable assessment of health status at the admission baseline (determined primarily by age, Charlson comorbidity, oxygenation/respiratory status and blood lab biomarkers) tended to experience worse outcomes. In this regard, phenotype *C* was linked to the most acute pathophysiologic presentations.

Overall, in terms of the 18 highlighted variables shown in Table 3, we deem our results to be coherent and in good agreement with the literature. Nonetheless, we found it unexpected –to a certain extent– that the general-purpose severity scores for community-acquired pneumonia (PSI, CURB-65) stood out as relevant. With the breakout of the COVID-19 pandemic, many works contributed with *ad hoc* scores which outperformed these.

Sex is another variable for which we observed a disparity of findings. In [2], the authors reported females to be overrepresented in their subphenotype I (low risk) and extra males in II (moderate risk) and IV (worst prognoses). Likewise, in [3–5] there were more women than average in their corresponding favourable phenotypes. Contrarily, in [6] the authors documented men to be underrepresented in the cluster with the highest in-hospital mortality. In the other works, sex-based differences were not emphasized as an important explanatory variable for differences across phenotypes; which was the case here.

Various factors with an influential role in the phenotypes from the literature were not available to us, after discarding variables with $\ge$60% missing measurements: platelets [1, 8], albumin [1, 2], ferritin [2, 9], alanine ALT [2, 6], interleukin IL-6 [2], bilirubin [8]. Besides, LDH was found to be strongly relevant in [3, 8, 9]; whereas here the inter-phenotype differences in terms of LDH were indeed statistically significant ($p\ll$0.001), although with a Kruskal-Wallis effect size $\eta _H^2$=0.117 of ‘only’ medium magnitude [21]. The inter-variable correlations when computing KPCA projection may explain –at least to a certain extent– this minor difference with respect to the literature.

On the other hand, our study has several limitations. First, its observational design may have introduced bias or residual confounding. A certain degree of inclusion bias may also be present due to the admission policies at Hospital III: forced by the unprecedented situation of the pandemic in the healthcare system, and given that this institution had many more ICU beds available than other local hospitals, patients triaged in emergencies as the most fragile or deteriorated were preferentially referred there. Besides, we could not provide information on underlying immunologic or virological mechanisms.

Our analyses here account only for baseline data: available at the time of admission, and up to 24 hours later. Thus, they incorporate neither disease dynamics nor the response to therapy, and we cannot ascertain whether or not this information may have affected phenotype delineation.

We did not have access to any individualized data concerning social determinants of health [2, 34]. Instead, socioeconomic information per postcode of residence could have been obtained from census data. However, here we opted for not following such an approximation, as it arguably would imply an additional source of bias: all patients living in a given postcode would always share common socioeconomic characterizations. The same reasoning applied to data about exposure to outdoor air pollution, despite the growing evidence in the literature on the role of air pollution in COVID-19 [35–39].

Another limitation consists in that the cohort belongs to the first wave of the COVID-19 pandemic in Spain (from February to May 2020). With such a choice, we aimed at learning patterns from patients who underwent the disease in a situation as uniform as possible: regarding the clinical knowledge available about COVID-19 and its treatment, and in terms of the burden to the healthcare system. Arguably, this first-wave situation can also have been detrimental to data collection: explaining, to an important extent, the high rates of data missingness.

In this regard, we deem it interesting for further research to investigate the algorithmic adaptations needed by the unsupervised clustering models for phenotype extraction, in order to accommodate datasets with time-induced distributional shifts [40, 41] (i.e. trends changing across pandemic waves); as well as on the external validation of the phenotypes with cohorts from different waves and locations.

## Conclusions

Prospective study exploring the existence of various clinical phenotypes in a cohort of hospitalized patients with SARS-CoV-2 pneumonia. Using unsupervised machine learning techniques (clustering), three distinct phenotypes were automatically extracted in our training set, which in turn generalized satisfactorily to the test set. Statistical analyses on the odds ratios for clinical severity and mortality revealed strongly significant differences across the three phenotypes in terms of both outcomes, pointing out the practical relevance of the phenotypes found. Furthermore, 18 out of 92 clinical explanatory variables showed large effect sizes with respect to the clustered cases, hence behaving as relevant factors for phenotype interpretation.

The identification of these phenotypes may aid clinicians in the early identification and characterization of patients for enhanced evidence-based clinical management, although the underlying pathophysiological mechanisms of the phenotypes should be further investigated. Among a population of highly heterogeneous hospitalized patients with SARS-CoV-2, profiling (via phenotypes) which subgroup is the most likely to deteriorate may contribute to initiating personalized treatments with a targeted therapeutic regime. Future research should ascertain the generalizability for cohorts from other locations and/or COVID-19 pandemic waves.

## Supplementary Information

**Additional file 1.** The online version contains supplementary material available at INSERT LINK HERE.

## Data Availability

No datasets were generated or analysed during the current study.
