# Supplementary material for: Obtaining patient phenotypes in SARS-CoV-2 pneumonia, and their association with clinical severity and mortality
Source: Pneumonia (Nathan). 2024 Jun 25;16:12. doi: 10.1186/s41479-024-00132-0 (PMC11637184; doi:10.1186/s41479-024-00132-0)
Supplement: Supplementary file 1 — Additional file 1. The online version contains supplementary material available at INSERT LINK HERE. [file 41479_2024_132_MOESM1_ESM.pdf]

On-line Supplementary Materials

A Descriptive of the cohort

Abbreviations for Table 1 – *NA*: not available, *IQR*: inter-quartile range, *K-W*: Kruskal-Wallis, *NS*: not significant, *BMI*: body-mass index, *ILD*: interstitial lung disease, *COPD*: chronic obstructive pulmonary disease, *HIV*: human immunodeficiency virus, *PSI*: pneumonia severity index, *CURB-65*: pneumonia severity score (confusion, urea, respiratory rate, blood pressure, age 65), *qSOFA*: quick sequential organ failure assessment score, *ACEI*: angiotensin-converting-enzyme inhibitors, *AIIRB*: angiotensin II receptor blockers, *BUN*: blood urea nitrogen, *GGT*: gamma-glutamyl transferase, *LDH*: lactate dehydrogenase, *CRP*: C-reactive protein, *PCT*: procalcitonin, *PCR*: polymerase chain reaction, *IV*: intravenous, *LMWH*: low molecular weight heparin.

Table 1: **Characteristics of our cohort, overall and by phenotype**

Variable		**Overall** n=1548	**By phenotype** ***Pheno A*** n=788 (50.9%)	***Pheno B*** n=620 (40.0%)	***Pheno C*** n=140 (9.0%)	p-value	Effect size	
Sex	Male	952 (61.5%)	441 (56.0%)	414 (66.8%)	97 (69.3%)	χ^2	V	
	Female	596 (38.5%)	347 (44.0%)	206 (33.2%)	43 (30.7%)			
	NA	0	0	0	0	<0.001	0.111	Small
Age [years]	Median	65	57	75	71	K-W	η^2_H	
	IQR	[53, 77]	[46, 66]	[66, 83]	[59, 82]			
	Num. valid	1548 (100%)	788 (100%)	620 (100%)	140 (100%)	<0.001	0.284	Large
Height [m]	Median	1.66	1.66	1.67	1.67	K-W	η^2_H	
	IQR	[1.60, 1.73]	[1.60, 1.73]	[1.60, 1.73]	[1.61, 1.72]			
	Num. valid	895 (57.8%)	446 (56.6%)	364 (58.2%)	85 (60.7%)	0.922	NS	NS
Weight [kg]	Median	78.5	79.0	77.0	85.0	K-W	η^2_H	
	IQR	[68.0, 89.0]	[68.0, 89.0]	[67.0, 87.0]	[75.3, 97.5]			
	Num. valid	982 (63.4%)	498 (63.2%)	394 (63.5%)	90 (64.3%)	<0.001	0.015	Small
Body mass index (BMI) $[kg/m^2]$	Median	27.78	27.77	27.34	30.28	K-W	η^2_H	
	IQR	[25.23, 31.55]	[25.00, 31.56]	[25.26, 30.97]	[26.91, 34.32]			
	Num. valid	866 (55.9%)	437 (55.5%)	346 (55.8%)	83 (59.3%)	<0.001	0.018	Small
Obesity	No	664 (42.9%)	333 (42.3%)	288 (46.5%)	43 (30.7%)	χ^2	V	
	Yes	343 (22.2%)	172 (21.8%)	125 (20.2%)	46 (32.9%)			
	NA	541 (34.9%)	283 (35.9%)	207 (33.4%)	51 (36.4%)	<0.001	0.113	Small
Alcohol use	No	941 (60.8%)	548 (69.5%)	323 (52.1%)	70 (50.0%)	χ^2	V	
	Ex	16 (1.0%)	7 (0.9%)	8 (1.3%)	1 (0.7%)			
	Yes	54 (3.5%)	18 (2.3%)	31 (5.0%)	5 (3.6%)			
	NA	537 (34.7%)	215 (27.3%)	258 (41.6%)	64 (45.7%)	0.005	0.073	Small
Tobacco use	No	900 (58.1%)	514 (65.2%)	310 (50.0%)	76 (54.3%)	χ^2	V	
	Ex	347 (22.4%)	165 (20.9%)	157 (25.3%)	25 (17.9%)			
	Yes	83 (5.4%)	48 (6.1%)	31 (5.0%)	4 (2.9%)			
	NA	218 (14.1%)	61 (7.7%)	122 (19.7%)	35 (25.0%)	0.008	0.060	Negligible
Lives at nursing home	No	1274 (82.3%)	724 (91.9%)	458 (73.9%)	92 (65.7%)	χ^2	V	
	Yes	94 (6.1%)	19 (2.4%)	58 (9.4%)	17 (12.1%)			
	NA	180 (11.6%)	45 (5.7%)	104 (16.8%)	31 (22.1%)	<0.001	0.187	Small
Comorbidity: Hypertension	No	801 (51.7%)	571 (72.5%)	173 (27.9%)	57 (40.7%)	χ^2	V	
	Yes	474 (48.3%)	217 (27.5%)	447 (72.1%)	83 (59.3%)			
	NA	0	0	0	0	<0.001	0.427	Medium
Comorbidity: Diabetes mellitus	No	1218 (78.7%)	710 (90.1%)	414 (66.8%)	94 (67.1%)	χ^2	V	
	Yes	326 (21.1%)	76 (9.6%)	204 (32.9%)	46 (32.9%)			
	NA	4 (0.3%)	2 (0.3%)	2 (0.3%)	0	<0.001	0.283	Small
Comorbidity: Dyslipidemia	No	973 (62.9%)	580 (73.6%)	303 (48.9%)	90 (64.3%)	χ^2	V	
	Yes	575 (37.1%)	208 (26.4%)	317 (51.1%)	50 (35.7%)			
	NA	0	0	0	0	<0.001	0.240	Small
Comorbidity: Cardiovascular	No	1264 (81.7%)	738 (93.7%)	419 (67.6%)	107 (76.4%)	χ^2	V	
	Yes	284 (18.3%)	50 (6.4%)	201 (32.4%)	33 (23.6%)			
	NA	0	0	0	0	<0.001	0.320	Medium
Comorbidity: Chronic heart failure	No	1111 (71.8%)	609 (77.3%)	404 (65.2%)	98 (70.0%)	χ^2	V	
	Yes	78 (5.0%)	8 (1.0%)	61 (9.8%)	9 (6.4%)			
	NA	359 (23.2%)	171 (21.7%)	155 (25.0%)	33 (23.6%)	<0.001	0.223	Small
Comorbidity: Cerebrovascular	No	1058 (68.3%)	589 (74.7%)	378 (61.0%)	91 (65.0%)	χ^2	V	
	Yes	132 (8.5%)	28 (3.6%)	88 (14.2%)	16 (11.4%)			
	NA	358 (23.1%)	171 (21.7%)	154 (24.8%)	33 (23.6%)	<0.001	0.215	Small

Variable		Overall	By phenotype			p-value	Effect size	
			Pheno A	***Pheno B***	***Pheno C***			
		n=1548	n=788 (50.9%)	n=620 (40.0%)	n=140 (9.0%)			
Comorbidity: Bronchopathy	No	1232 (79.6%)	649 (82.4%)	472 (76.1%)	111 (79.3%)	χ^2	V	
	Asthma	87 (5.6%)	63 (8.0%)	18 (2.9%)	6 (4.3%)			
	ILD	5 (0.3%)	0	5 (0.8%)	0			
	COPD	102 (6.6%)	27 (3.4%)	66 (10.6%)	9 (6.4%)			
	Other	122 (7.9%)	49 (6.2%)	59 (9.5%)	14 (10.0%)			
	NA	0	0	0	0	<0.001	0.128	Small
Comorbidity: Renal	No	1356 (87.6%)	769 (97.6%)	468 (75.5%)	119 (85.0%)	χ^2	V	
	Yes	186 (12.0%)	18 (2.3%)	150 (24.2%)	18 (12.9%)			
	NA	6 (0.4%)	1 (0.1%)	2 (0.3%)	3 (2.1%)	<0.001	0.318	Medium
Comorbidity: Hepatic	No	1496 (96.6%)	772 (98.0%)	588 (94.8%)	136 (97.1%)	χ^2	V	
	Yes	52 (3.4%)	16 (2.0%)	32 (5.2%)	4 (2.9%)			
	NA	0	0	0	0	0.005	0.075	Negligible
Comorbidity: Ischemia	No	1064 (68.7%)	599 (76.0%)	384 (61.9%)	81 (57.9%)	χ^2	V	
	Yes	46 (3.0%)	9 (1.1%)	34 (5.5%)	3 (2.1%)			
	NA	438 (28.3%)	180 (22.8%)	202 (32.6%)	56 (40.0%)	<0.001	0.152	Small
Comorbidity: Peptic ulcer	No	1086 (70.2%)	602 (76.4%)	404 (65.2%)	80 (57.1%)	χ^2	V	
	Yes	24 (1.6%)	6 (0.8%)	14 (2.3%)	4 (2.9%)			
	NA	438 (28.3%)	180 (22.8%)	202 (32.6%)	56 (40.0%)	0.009	0.082	Negligible
Comorbidity: Thyroid	No	664 (42.9%)	342 (43.4%)	269 (43.4%)	53 (37.9%)	χ^2	V	
	Yes	73 (4.7%)	41 (5.2%)	28 (4.5%)	4 (2.9%)			
	NA	811 (52.4%)	405 (51.4%)	323 (52.1%)	83 (59.3%)	0.643	NS	NS
Comorbidity: Autoimmune	No	1120 (72.4%)	543 (68.9%)	467 (75.3%)	110 (78.6%)	χ^2	V	
	Yes	52 (3.4%)	20 (2.5%)	29 (4.7%)	3 (2.1%)			
	NA	376 (24.3%)	225 (28.6%)	124 (20.0%)	27 (19.3%)	0.122	NS	NS
Comorbidity: Transplants	No	1094 (70.7%)	604 (76.6%)	407 (65.6%)	83 (59.3%)	χ^2	V	
	Yes	16 (1.0%)	4 (0.5%)	11 (1.8%)	1 (0.7%)			
	NA	438 (28.3%)	180 (22.8%)	202 (32.6%)	56 (40.0%)	0.033	0.066	Negligible
Comorbidity: Cancer, neoplasia	No	1428 (92.2%)	760 (96.4%)	539 (86.9%)	129 (92.1%)	χ^2	V	
	Yes	120 (7.8%)	28 (3.6%)	81 (13.1%)	11 (7.9%)			
	NA	0	0	0	0	<0.001	0.165	Small
Comorbidity: Immune, HIV	No	1431 (92.4%)	759 (96.3%)	546 (88.1%)	126 (90.0%)	χ^2	V	
	Yes	67 (4.3%)	12 (1.5%)	49 (7.9%)	6 (4.3%)			
	NA	50 (3.2%)	17 (2.2%)	25 (4.0%)	8 (5.7%)	<0.001	0.149	Small
Comorbidity: Overall	No	381 (24.6%)	315 (40.0%)	41 (6.6%)	25 (17.9%)	χ^2	V	
	Yes	1167 (75.4%)	473 (60.0%)	579 (93.4%)	115 (82.1%)			
	NA	0	0	0	0	<0.001	0.368	Medium
Comorbidity: Charlson index	Median	3	1	5	4	K-W	η^2_H	
	IQR	[1, 5]	[0, 3]	[3, 7]	[2, 6]			
	Num. valid	1548 (100%)	788 (100%)	620 (100%)	140 (100%)	<0.001	0.351	Large
Pneumonia: PSI score	Median	70	55	91	95	K-W	η^2_H	
	IQR	[53, 92]	[45, 68]	[76, 114]	[76, 114]			
	Num. valid	1287 (83.1%)	705 (89.5%)	479 (77.3%)	103 (73.6%)	<0.001	0.433	Large
Pneumonia: CURB-65 score	0	539 (34.8%)	468 (59.4%)	49 (7.9%)	22 (15.7%)	χ^2	V	
	1	516 (33.3%)	257 (32.6%)	218 (35.2%)	41 (29.3%)			
	2	361 (23.3%)	50 (6.3%)	261 (42.1%)	50 (35.7%)			
	3	89 (5.7%)	4 (0.5%)	66 (10.6%)	19 (13.6%)			
	4	20 (1.3%)	0	12 (1.9%)	8 (5.7%)			
	NA	23 (1.5%)	9 (1.1%)	14 (2.3%)	0	<0.001	0.439	Large
Sepsis: qSOFA score	0	791 (51.1%)	518 (65.7%)	252 (40.6%)	21 (15.0%)	χ^2	V	
	1	274 (17.7%)	87 (11.0%)	140 (22.6%)	47 (33.6%)			

Variable		**Overall**	**By phenotype** *Pheno A*	*Pheno B*	*Pheno C*			
		n=1548	n=788 (50.9%)	n=620 (40.0%)	n=140 (9.0%)	p-value	Effect size	
	2	39 (2.5%)	3 (0.4%)	22 (3.5%)	14 (10.0%)			
	3	3 (0.2%)	0	1 (0.2%)	2 (1.4%)			
	NA	441 (28.5%)	180 (22.8%)	205 (33.1%)	56 (40.0%)	<0.001	0.295	Medium
Symptoms: Cough	No	404 (26.1%)	177 (22.5%)	190 (30.6%)	37 (26.4%)	χ^2	V	
	Yes	1015 (65.6%)	582 (73.9%)	354 (57.1%)	79 (56.4%)			
	NA	129 (8.3%)	29 (3.7%)	76 (12.3%)	24 (17.1%)	<0.001	0.118	Small
Symptoms: Expectoration	No	1166 (75.3%)	629 (79.8%)	438 (70.6%)	99 (70.7%)	χ^2	V	
	Yes	254 (16.4%)	131 (16.6%)	106 (17.1%)	17 (12.1%)			
	NA	128 (8.3%)	28 (3.6%)	76 (12.3%)	24 (17.1%)	0.370	NS	NS
Symptoms: Dyspnea	No	754 (48.7%)	434 (55.1%)	289 (46.6%)	31 (22.1%)	χ^2	V	
	Yes	669 (43.2%)	327 (41.5%)	257 (41.5%)	85 (60.7%)			
	NA	125 (8.1%)	27 (3.4%)	74 (11.9%)	24 (17.1%)	<0.001	0.157	Small
Symptoms: Myalgia	No	1108 (71.6%)	558 (70.8%)	451 (72.7%)	99 (70.7%)	χ^2	V	
	Yes	314 (20.3%)	203 (25.8%)	94 (15.2%)	17 (12.1%)			
	NA	126 (8.1%)	27 (3.4%)	75 (12.1%)	24 (17.1%)	<0.001	0.114	Small
Symptoms: Confusion	No	1336 (86.3%)	750 (95.2%)	485 (78.2%)	101 (72.1%)	χ^2	V	
	Yes	86 (5.6%)	11 (1.4%)	59 (9.5%)	16 (11.4%)			
	NA	126 (9.1%)	27 (3.4%)	76 (12.3%)	23 (16.4%)	<0.001	0.206	Small
Symptoms: Thorax pain	No	1279 (82.6%)	674 (85.5%)	495 (79.8%)	110 (78.6%)	χ^2	V	
	Yes	143 (9.2%)	87 (11.0%)	50 (8.1%)	6 (4.3%)			
	NA	126 (8.1%)	27 (3.4%)	75 (12.1%)	24 (17.1%)	0.077	NS	NS
Symptoms: Anosmia	No	1320 (85.3%)	620 (78.7%)	571 (92.1%)	129 (92.1%)	χ^2	V	
	Yes	219 (14.1%)	165 (20.9%)	43 (6.9%)	11 (7.9%)			
	NA	9 (0.6%)	3 (0.4%)	6 (1.0%)	0	<0.001	0.195	Small
Symptoms: Fever	No	235 (15.2%)	103 (13.1%)	112 (18.1%)	20 (14.3%)	χ^2	V	
	Febricula	272 (17.6%)	137 (17.4%)	116 (18.7%)	19 (13.6%)			
	Fever	916 (59.2%)	521 (66.1%)	318 (51.3%)	77 (55.0%)			
	NA	125 (8.1%)	27 (3.4%)	74 (11.9%)	24 (17.2%)	0.002	0.067	Small
Symptoms: Digestive-gastro	No	1053 (68.0%)	541 (68.6%)	420 (67.7%)	92 (65.7%)	χ^2	V	
	Yes	353 (22.8%)	210 (26.6%)	119 (19.2%)	24 (17.1%)			
	NA	142 (9.2%)	37 (4.7%)	81 (13.1%)	24 (17.1%)	0.029	0.060	Negligible
Symptoms: Overall	No	1 (0.1%)	0	1 (0.2%)	0	χ^2	V	
	Yes	1547 (99.9%)	788 (100%)	619 (99.8%)	140 (100%)			
	NA	0	0	0	0	0.473	NS	NS
Symptoms: Days	Median	7	7	6	7			
	IQR	[4, 10]	[5, 10]	[3, 9]	[5, 9]	K-W	η^2_H	
	Num. valid	1415 (91.4%)	758 (96.2%)	541 (87.3%)	116 (82.9%)	<0.001	0.024	Small
Emergency treatm: ACEI-AIIRB	No	745 (45.1%)	487 (61.8%)	210 (33.9%)	48 (34.3%)	χ^2	V	
	Yes	365 (23.6%)	121 (15.4%)	208 (33.5%)	36 (25.7%)			
	NA	438 (28.3%)	180 (22.8%)	202 (32.6%)	56 (40.0%)	<0.001	0.304	Medium
Emergency treatm: Statin	No	960 (62.0%)	610 (77.4%)	270 (43.5%)	80 (57.1%)	χ^2	V	
	Yes	410 (26.5%)	135 (17.1%)	246 (39.7%)	29 (20.7%)			
	NA	178 (11.5%)	43 (5.5%)	104 (16.8%)	31 (22.1%)	<0.001	0.303	Medium
Emergency treatm: Anticoagulant	No	1406 (90.8%)	765 (97.1%)	511 (82.4%)	130 (92.9%)	χ^2	V	
	Yes	142 (9.2%)	23 (2.9%)	109 (17.6%)	10 (7.1%)			
	NA	0	0	0	0	<0.001	0.239	Small
Emergency treatm: Antiplatelet	No	980 (63.3%)	577 (73.2%)	328 (52.9%)	75 (53.6%)	χ^2	V	
	Yes	165 (10.7%)	41 (5.2%)	112 (18.1%)	12 (8.6%)			
	NA	403 (26.0%)	170 (21.6%)	180 (29.0%)	53 (37.9%)	<0.001	0.251	Small

Variable		Overall n=1548	By phenotype ***Pheno A*** n=788 (50.9%)	***Pheno B*** n=620 (40.0%)	***Pheno C*** n=140 (9.0%)	p-value	Effect size	
Emergency treatm: Corticosteroids	No	1180 (76.2%)	665 (84.4%)	418 (11.3%)	97 (69.3%)	χ^2	V	
	Inhaled	145 (9.4%)	67 (8.5%)	70 (67.4%)	8 (5.7%)			
	Oral	44 (2.8%)	13 (1.6%)	27 (4.4%)	4 (2.9%)			
	NA	179 (11.6%)	43 (5.5%)	105 (16.9%)	31 (22.1%)	<0.001	0.080	Small
Admission status: Body temperature	Median	37.0	37.0	37.0	37.1	K-W	η^2_H	
[$°C$]	IQR	[36.4, 37.7]	[36.4, 37.8]	[36.4, 37.8]	[36.5, 37.8]			
	Num. valid	1347 (87.0%)	743 (94.3%)	507 (81.8%)	97 (69.3%)	0.639	NS	NS
Admission status: Systolic blood	Median	127	126	128	120	K-W	η^2_H	
pressure [$mmHg$]	IQR	[115, 142]	[116, 139]	[115, 146]	[107, 139]			
	Num. valid	1339 (86.5%)	740 (93.9%)	501 (80.8%)	98 (70.0%)	0.003	0.007	Negligible
Admission status: Diastolic blood	Median	75	78	73	70	K-W	η^2_H	
pressure [$mmHg$]	IQR	[68, 83]	[70, 85]	[65, 81]	[63, 78]			
	Num. valid	1338 (86.4%)	739 (93.8%)	501 (80.8%)	98 (70.0%)	<0.001	0.046	Small
Admission status: Respiratory rate	Median	18	18	20	30	K-W	η^2_H	
[min^{-1}]	IQR	[16, 24]	[16, 20]	[17, 25]	[23, 32]			
	Num. valid	1021 (66.0%)	577 (73.2%)	364 (58.7%)	80 (57.1%)	<0.001	0.149	Large
Admission status: Heart rate	Median	90	92	88	90	K-W	η^2_H	
[min^{-1}]	IQR	[80, 102]	[80, 104]	[77, 100]	[84, 95]			
	Num. valid	1525 (98.5%)	784 (99.5%)	608 (98.0%)	133 (95.0%)	<0.001	0.013	Small
Admission status: SpO_2 [%]	Median	95	96	94	90	K-W	η^2_H	
	IQR	[93, 97]	[94, 97]	[91, 96]	[84, 95]			
	Num. valid	1529 (98.8%)	785 (99.6%)	609 (98.2%)	135 (96.4%)	<0.001	0.129	Medium
Admission status: FiO_2 [$fraction$]	Median	0.21	0.21	0.21	0.38	K-W	η^2_H	
	IQR	[0.21, 0.21]	[0.21, 0.21]	[0.21, 0.21]	[0.21, 0.83]			
	Num. valid	1526 (98.6%)	782 (99.2%)	608 (98.0%)	136 (97.1%)	<0.001	0.420	Large
Admission status: SpO_2/FiO_2	Median	452.38	457.14	447.62	238.10	K-W	η^2_H	
[$ratio$]	IQR	[433.33, 461.90]	[447.62, 461.90]	[423.81, 457.14]	[105.63, 317.86]			
	Num. valid	1520 (98.2%)	781 (99.1%)	604 (97.4%)	135 (96.4%)	<0.001	0.300	Large
Admission status: $SpO_2/RespRate$	Median	5.17	5.44	4.70	2.97	K-W	η^2_H	
[$\%/min^{-1}$]	IQR	[4.00, 5.94]	[4.75, 6.00]	[3.65, 5.56]	[2.29, 3.92]			
	Num. valid	1017 (65.7%)	577 (73.2%)	363 (58.5%)	77 (55.0%)	<0.001	0.178	Large
Pulmonary status: Crackles	No	390 (25.2%)	228 (28.9%)	141 (22.7%)	21 (15.0%)	χ^2	V	
	Yes	601 (38.8%)	293 (37.2%)	251 (40.5%)	57 (40.7%)			
	NA	557 (36.0%)	267 (33.9%)	228 (33.8%)	62 (44.3%)	0.004	0.096	Negligible
Pulmonary status: Infiltr. X-Ray	No	116 (7.5%)	52 (6.6%)	62 (10.0%)	2 (1.4%)	χ^2	V	
	Unilobar	227 (14.7%)	161 (20.4%)	56 (9.0%)	10 (7.1%)			
	Multilob unilat	65 (4.2%)	34 (4.3%)	27 (4.4%)	4 (2.9%)			
	Bilateral	701 (45.3%)	361 (45.8%)	272 (43.9%)	68 (48.6%)			
	NA	439 (28.4%)	180 (22.8%)	203 (32.7%)	50 (40.0%)	<0.001	0.133	Small
Pulmonary status: Infiltr. type	No	117 (7.6%)	53 (6.7%)	62 (10.0%)	2 (1.4%)	χ^2	V	
	Alveolar	334 (21.6%)	179 (22.7%)	127 (20.5%)	28 (20.0%)			
	Interstitial	770 (49.7%)	419 (53.2%)	279 (45.0%)	72 (51.4%)			
	Consolidation	163 (10.5%)	90 (11.4%)	63 (10.2%)	10 (7.1%)			
	NA	164 (10.6%)	47 (6.0%)	89 (14.4%)	28 (20.0%)	0.008	0.064	Negligible
Pulmonary status: Infiltration,	0	123 (7.9%)	54 (6.9%)	66 (10.6%)	3 (2.1%)	χ^2	V	
Num. lobes	1	253 (16.3%)	176 (22.3%)	69 (11.1%)	8 (5.7%)			
	2	406 (26.2%)	235 (29.8%)	151 (24.4%)	20 (14.3%)			
	3	205 (13.2%)	113 (14.3%)	76 (12.3%)	16 (11.4%)			
	4	188 (12.1%)	95 (12.1%)	68 (11.0%)	25 (17.9%)			
	5	119 (7.7%)	41 (5.2%)	54 (8.7%)	24 (17.1%)			

Variable		**Overall** n=1548	**By phenotype** ***Pheno A*** n=788 (50.9%)	***Pheno B*** n=620 (40.0%)	***Pheno C*** n=140 (9.0%)	p-value	Effect size	
	6	49 (3.2%)	20 (2.6%)	21 (3.4%)	8 (5.7%)			
	NA	205 (13.2%)	54 (6.9%)	115 (18.5%)	36 (25.7%)	<0.001	0.175	Small
Pulmonary status: Pleural effusion	No	1317 (85.1%)	731 (92.8%)	482 (77.7%)	104 (74.3%)	χ^2	V	
	Yes	37 (2.4%)	11 (1.4%)	22 (3.5%)	17 (2.9%)			
	NA	194 (12.5%)	46 (5.8%)	116 (18.7%)	116 (22.9%)	0.007	0.076	Negligible
Blood test: Glucose [mg/dL]	Median	111	104	123	133	K-W	η^2_H	
	IQR	[99, 134]	[96, 119]	[108, 159]	[106, 181]			
	Num. valid	1383 (89.3%)	751 (95.3%)	524 (84.5%)	108 (77.1%)	<0.001	0.134	Medium
Blood test: Urea [mg/dL]	Median	34	28	48	48	K-W	η^2_H	
	IQR	[26, 47]	[22, 33]	[38, 64]	[35, 78]			
	Num. valid	1107 (71.5%)	606 (76.9%)	418 (67.4%)	83 (59.3%)	<0.001	0.411	Large
Blood test: Creatinine [mg/dL]	Median	0.92	0.82	1.09	1.04	K-W	η^2_H	
	IQR	[0.75, 1.13]	[0.69, 0.96]	[0.88, 1.50]	[0.84, 1.44]			
	Num. valid	1476 (95.3%)	763 (96.8%)	585 (94.4%)	128 (91.4%)	<0.001	0.213	Large
Blood test: Blood urea nitrogen (BUN) [mg/dL]	Median	17.0	15.0	20.3	20.0	K-W	η^2_H	
	IQR	[13.0, 23.0]	[12.0, 20.0]	[15.0, 27.0]	[15.0, 28.5]			
	Num. valid	736 (47.5%)	382 (48.5%)	297 (47.9%)	57 (40.7%)	<0.001	0.390	Large
Blood: Sodium [mEq/L]	Median	138	138	137	138	K-W	η^2_H	
	IQR	[136, 140]	[136, 140]	[135, 140]	[136, 140]			
	Num. valid	1396 (90.2%)	753 (95.6%)	530 (85.5%)	113 (80.7%)	<0.001	0.017	Small
Blood test: Gamma-glutamyl transferase (GGT) [U/L]	Median	27	29	24	31	K-W	η^2_H	
	IQR	[18, 44]	[19, 46]	[16, 41]	[20, 53]			
	Num. valid	1265 (81.7%)	700 (88.8%)	466 (75.2%)	99 (70.7%)	0.001	0.012	Small
Blood test: Lactate dehydrogenase (LDH) [U/L]	Median	301.5	273.0	345.0	411.0	K-W	η^2_H	
	IQR	[238.0, 389.8]	[229.0, 333.3]	[254.0, 439.0]	[332.0, 560.0]			
	Num. valid	1162 (75.1%)	644 (81.7%)	429 (69.2%)	89 (63.6%)	<0.001	0.117	Medium
Blood test: C-reactive protein (CRP) [mg/L]	Median	72.13	49.51	101.63	152.60	K-W	η^2_H	
	IQR	[32.30, 134.04]	[22.99, 91.90]	[50.09, 162.21]	[96.82, 256.47]			
	Num. valid	1473 (95.2%)	759 (96.3%)	586 (94.5%)	128 (91.4%)	<0.001	0.167	Large
Blood test: Procalcitonin (PCT) [μg/L]	Median	0.11	0.07	0.18	0.25	K-W	η^2_H	
	IQR	[0.06, 0.22]	[0.04, 0.12]	[0.10, 0.44]	[0.12, 0.81]			
	Num. valid	1089 (70.3%)	549 (69.7%)	440 (71.0%)	100 (71.4%)	<0.001	0.285	Large
Blood test: Hematocrit [%]	Median	41.0	42.0	39.8	40.8	K-W	η^2_H	
	IQR	[37.7, 44.1]	[39.0, 44.5]	[36.0, 43.2]	[37.3, 44.0]			
	Num. valid	1371 (88.6%)	744 (94.4%)	520 (83.9%)	107 (76.4%)	<0.001	0.042	Small
Blood test: Leukocytes [count/μL]	Median	6240	5680	6955	7825	K-W	η^2_H	
	IQR	[4710, 8373]	[4400, 7148]	[5200, 9560]	[5760, 11735]			
	Num. valid	1460 (94.3%)	754 (95.7%)	580 (93.5%)	126 (90.0%)	<0.001	0.069	Medium
Blood test: Lymphocytes [count/μL]	Median	900	1060	730	700	K-W	η^2_H	
	IQR	[640, 1240]	[800, 1330]	[500, 1058]	[500, 1088]			
	Num. valid	1530 (98.8%)	782 (99.2%)	610 (98.4%)	138 (98.6%)	<0.001	0.109	Medium
Blood test: Neutrophils [count/μL]	Median	4700	3955	5335	6500	K-W	η^2_H	
	IQR	[3300, 6620]	[2993, 5435]	[3878, 7623]	[4530, 10340]			
	Num. valid	1545 (99.8%)	786 (99.7%)	610 (98.4%)	138 (98.6%)	<0.001	0.107	Medium
Blood test: Monocytes [count/μL]	Median	420	410	430	450	K-W	η^2_H	
	IQR	[290, 590]	[300, 550]	[290, 630]	[275, 650]			
	Num. valid	1108 (71.6%)	607 (77.0%)	418 (67.4%)	83 (59.3%)	0.373	NS	NS
Blood test: Basophils [count/μL]	Median	20	20	20	20	K-W	η^2_H	
	IQR	[10, 20]	[10, 20]	[10, 30]	[10, 30]			
	Num. valid	684 (44.2%)	406 (51.5%)	231 (37.3%)	47 (33.6%)	0.026	0.008	Negligible

Variable		**Overall** n=1548	**By phenotype** ***Pheno A*** n=788 (50.9%)	***Pheno B*** n=620 (40.0%)	***Pheno C*** n=140 (9.0%)	p-value	Effect size	
Blood test: Neutrophils vs. lympho [ratio]	Median	4.98	3.83	7.13	9.68			
	IQR	[3.33, 8.61]	[2.72, 5.37]	[4.43, 11.75]	[5.57, 14.85]	K-W	η^2_H	
	Num. valid	1529 (98.8%)	781 (99.1%)	610 (98.4%)	138 (98.6%)	<0.001	0.218	Large
Blood test: Fibrinogen [mg/dL]	Median	700	663	700	700			
	IQR	[562, 744]	[524, 700]	[618, 750]	[667, 750]	K-W	η^2_H	
	Num. valid	654 (42.2%)	360 (45.7%)	244 (39.4%)	50 (35.7%)	<0.001	0.055	Small
Blood test: D-dimer [ng/mL]	Median	751	540	1079	1286			
	IQR	[430, 1340]	[350, 865]	[636, 2100]	[819, 2406]	K-W	η^2_H	
	Num. valid	1268 (81.9%)	679 (86.2%)	482 (77.7%)	107 (76.4%)	<0.001	0.185	Large
Blood test: Prothrombin index [%]	Median	93	99	84	91			
	IQR	[81, 100]	[89, 100]	[62, 96]	[81, 100]	K-W	η^2_H	
	Num. valid	662 (42.8%)	346 (43.9%)	264 (42.6%)	52 (37.1%)	<0.001	0.171	Large
Arterial blood gas test: $SatO_2$ [%]	Median	95	96	94	92			
	IQR	[93, 97]	[94, 97]	[91, 85]	[85, 95]	K-W	η^2_H	
	Num. valid	743 (48.0%)	382 (48.5%)	297 (47.9%)	64 (45.7%)	<0.001	0.119	Medium
Arterial blood gas test: FiO_2 [fraction]	Median	0.21	0.21	0.21	0.21			
	IQR	[0.21, 0.21]	[0.21, 0.21]	[0.21, 0.21]	[0.21, 0.80]	K-W	η^2_H	
	Num. valid	946 (61.1%)	474 (60.2%)	381 (61.5%)	91 (65.0%)	<0.001	0.199	Large
Arterial blood gas test: $SatO_2/FiO_2$ [ratio]	Median	452.38	457.14	442.86	270.00			
	IQR	[420.24, 461.90]	[447.62, 461.90]	[400.00, 457.14]	[124.64, 395.68]	K-W	η^2_H	
	Num. valid	730 (47.2%)	374 (47.5%)	294 (47.4%)	62 (44.3%)	<0.001	0.220	Large
COVID-19 diagn: Method	Rapid serology	50 (3.2%)	33 (4.2%)	14 (2.3%)	3 (2.1%)	χ^2	V	
	PCR sputum	27 (1.7%)	18 (2.3%)	7 (1.1%)	2 (1.4%)			
	PCR nasophar	880 (56.8%)	448 (56.9%)	361 (58.2%)	71 (50.7%)			
	NA	591 (38.2%)	289 (36.7%)	238 (38.4%)	64 (45.7%)	0.145	NS	NS
COVID-19 diagn: Days diagnosed before admission	Median	0	1	1	0			
	IQR	[0, 1]	[0, 1]	[0, 1]	[0, 3]	K-W	η^2_H	
	Num. valid	811 (52.4%)	420 (53.3%)	325 (52.4%)	66 (47.1%)	0.087	NS	NS
COVID-19 diagn: Antigens in urine	No	662 (42.8%)	340 (43.1%)	272 (43.9%)	50 (35.7%)	χ^2	V	
	Yes	42 (2.7%)	12 (1.5%)	21 (3.4%)	9 (6.4%)			
	NA	844 (54.5%)	436 (55.3%)	327 (52.7%)	81 (57.9%)	<0.001	0.130	Small
Emerg. COVID-19 treatm: Antibiotics	No	682 (44.1%)	312 (39.6%)	297 (47.9%)	73 (52.1%)	χ^2	V	
	Beta-lactam	230 (14.9%)	115 (14.6%)	96 (15.5%)	19 (13.6%)			
	Macrolides	89 (5.8%)	57 (7.2%)	29 (4.7%)	3 (2.1%)			
	Macrol & Beta	486 (31.4%)	278 (35.3%)	172 (27.7%)	36 (25.7%)			
	Quinolones	30 (1.9%)	17 (2.2%)	10 (1.6%)	3 (2.1%)			
	Others	31 (2.0%)	9 (1.1%)	16 (2.6%)	6 (4.3%)			
	NA	0	0	0	0	<0.001	0.083	Small
Emerg. COVID-19 treatm: Chloroquine	No	485 (31.3%)	188 (23.9%)	237 (38.2%)	60 (42.9%)	χ^2	V	
	Yes	1063 (68.7%)	600 (76.1%)	383 (61.8%)	80 (57.1%)			
	NA	0	0	0	0	<0.001	0.162	Small
Emerg. COVID-19 treatm: Kaletra	No	814 (52.6%)	377 (47.8%)	349 (56.3%)	88 (62.9%)	χ^2	V	
	Yes	734 (47.4%)	411 (52.2%)	271 (43.7%)	52 (37.1%)			
	NA	0	0	0	0	<0.001	0.097	Negligible
Emerg. COVID-19 treatm: Remdesivir	No	1537 (99.3%)	778 (98.7%)	619 (99.8%)	140 (100%)	χ^2	V	
	Yes	11 (0.7%)	10 (1.3%)	1 (0.2%)	0			
	NA	0	0	0	0	0.028	0.058	Negligible
Emerg. COVID-19 treatm: Interferon Beta-1a	No	1436 (92.8%)	743 (94.3%)	570 (91.9%)	123 (87.9%)	χ^2	V	
	Yes	112 (7.2%)	45 (5.7%)	50 (8.1%)	17 (12.1%)			

Variable		**Overall** n=1548	**By phenotype** ***Pheno A*** n=788 (50.9%)	***Pheno B*** n=620 (40.0%)	***Pheno C*** n=140 (9.0%)	p-value	Effect size	
	NA	0	0	0	0	0.015	0.064	Negligible
Emerg. COVID-19 treatm: IV corticoids	No	1162 (75.1%)	681 (86.4%)	404 (65.2%)	77 (55.0%)	χ^2	V	
	Low	43 (2.8%)	16 (2.0%)	24 (3.9%)	3 (2.1%)			
	High	105 (6.8%)	27 (3.4%)	59 (9.5%)	19 (13.6%)			
	NA	238 (15.4%)	64 (8.1%)	133 (21.5%)	41 (29.3%)	<0.001	0.139	Small
Emerg. COVID-19 treatm: LMWH	No	208 (13.4%)	108 (13.7%)	82 (13.%)	18 (12.9%)	χ^2	V	
	Prophylaxis	682 (44.1%)	441 (56.0%)	199 (32.1%)	42 (30.0%)			
	Below therap.	67 (4.3%)	21 (2.7%)	42 (6.8%)	4 (2.9%)			
	Therapeut.	94 (6.1%)	15 (1.9%)	66 (10.6%)	13 (9.3%)			
	High risk	58 (3.7%)	23 (2.9%)	28 (4.5%)	7 (5.0%)			
	NA	439 (28.4%)	180 (22.8%)	203 (32.7%)	56 (40.0%)	<0.001	0.213	Medium

Univariate statistical comparisons for discrete variables were performed by means of the χ^2 test, and bias-corrected Cramer's V effect size [1].
For continuous variables, univariate comparisons were made with the non-parametric Kruskal-Wallis test, and its corresponding η_H^2 effect size.
Thresholds for interpreting effect sizes were taken from [2].

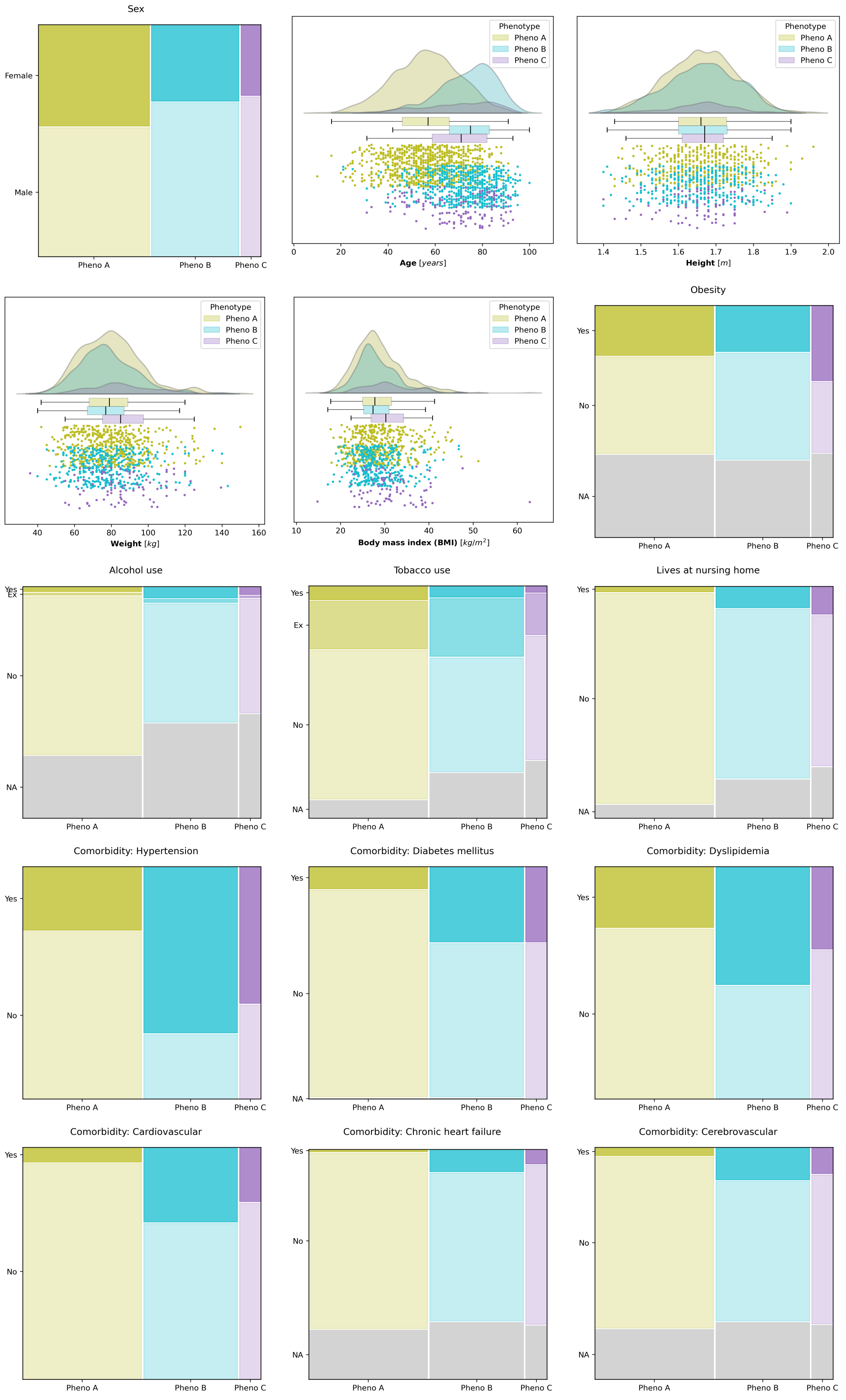

Fig. 1. Univariate descriptive graphics (1 of 7).

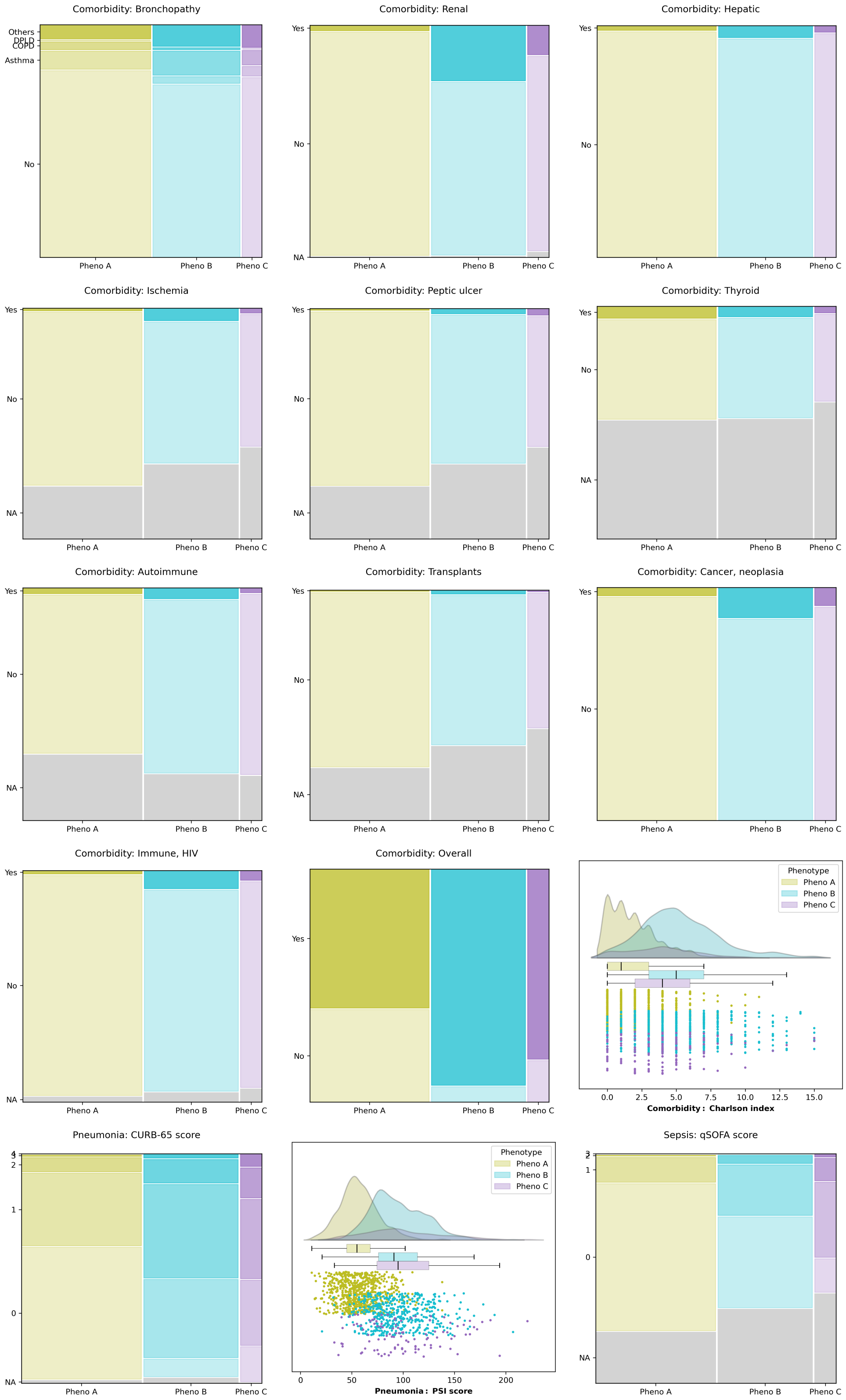

Fig. 2. Univariate descriptive graphics (2 of 7).

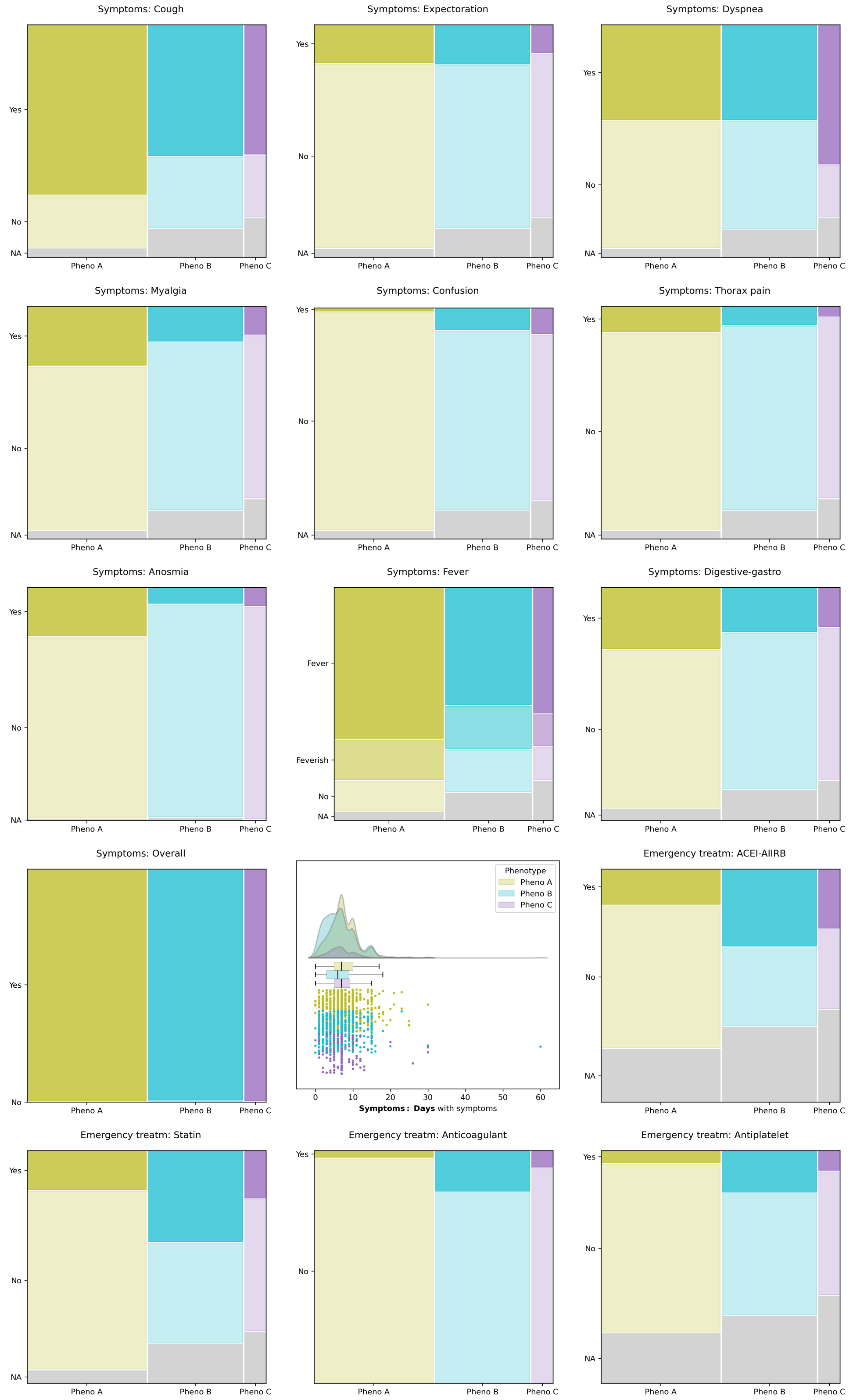

Fig. 3. Univariate descriptive graphics (3 of 7).

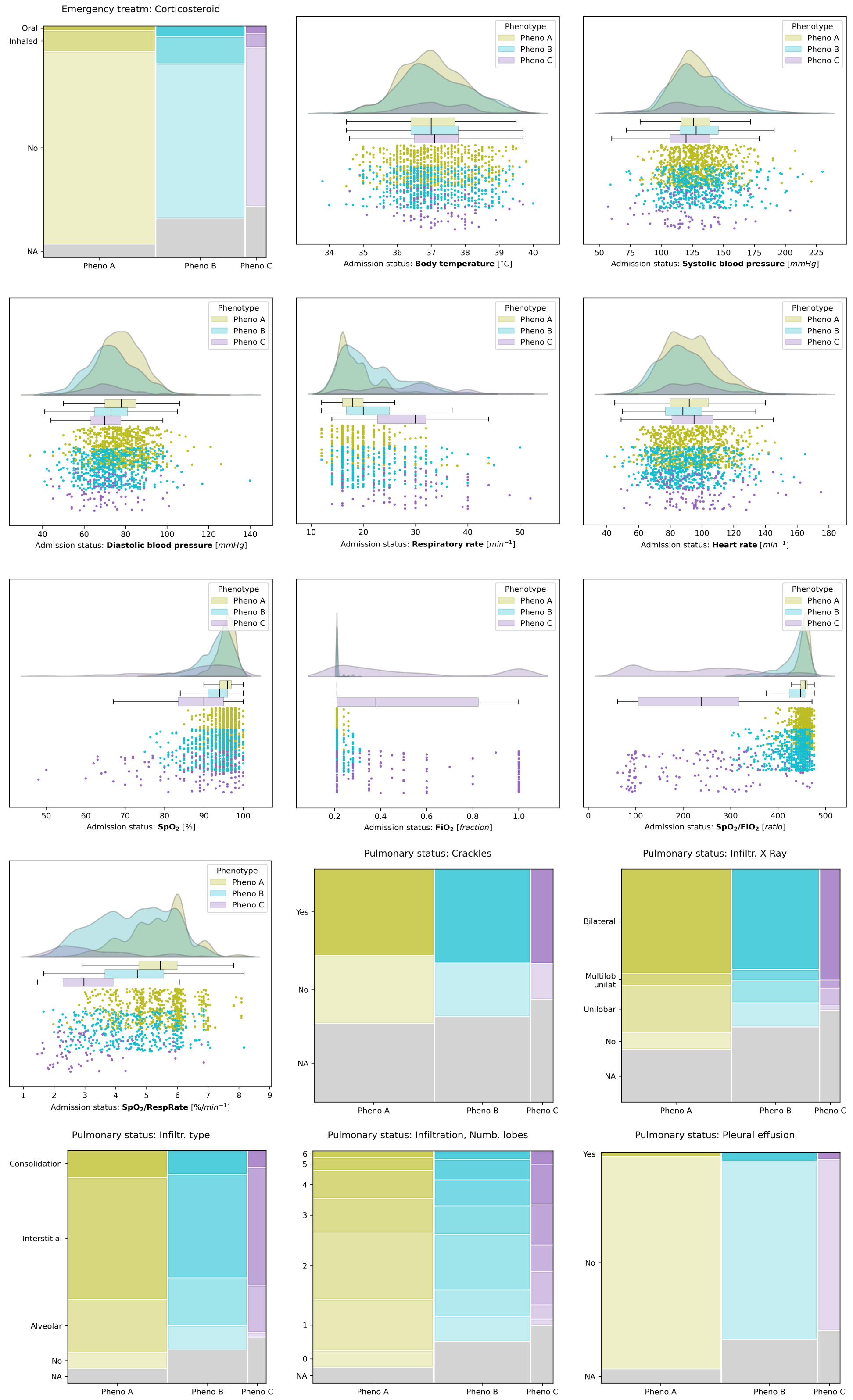

Fig. 4. Univariate descriptive graphics (4 of 7).

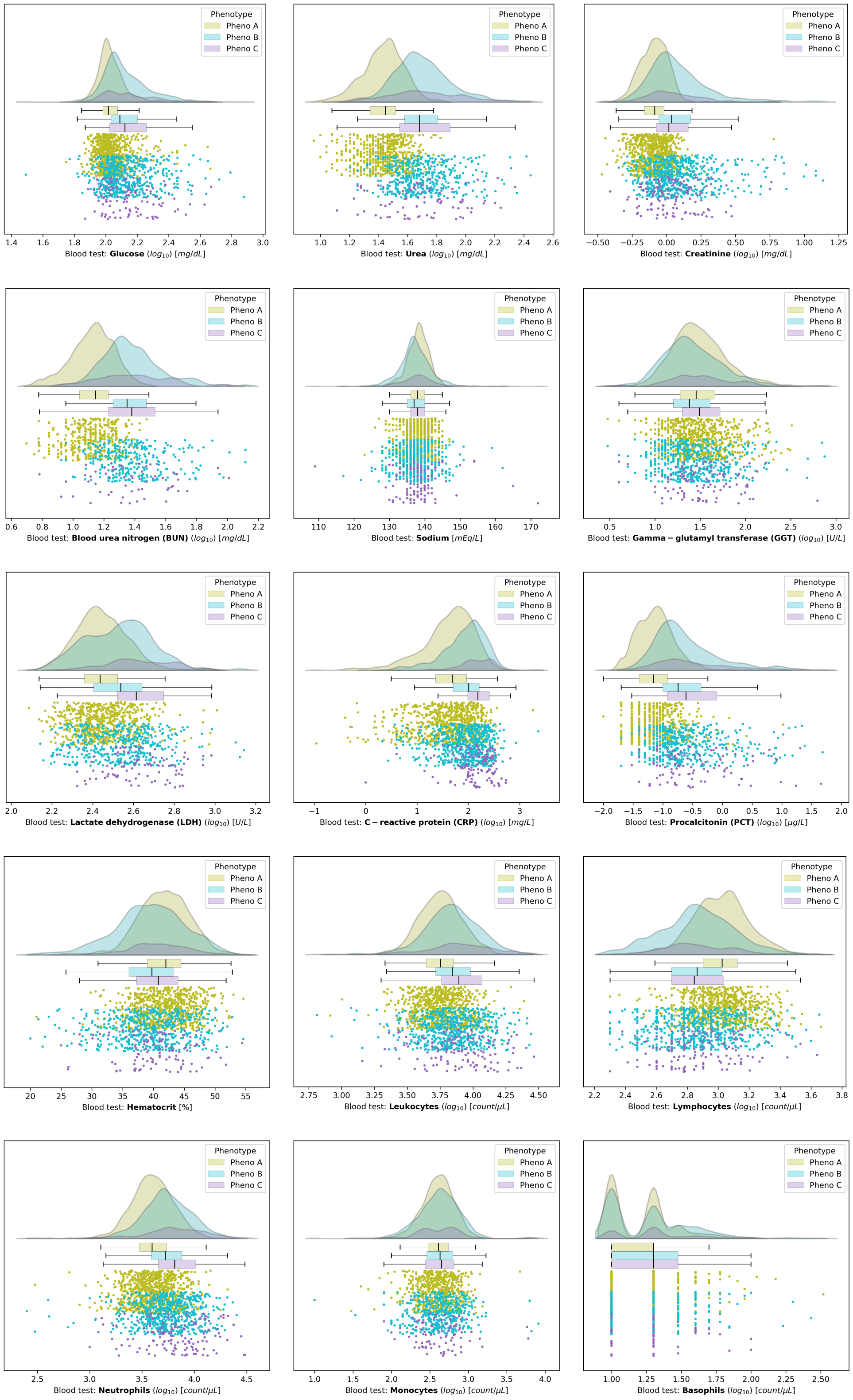

Fig. 5. Univariate descriptive graphics (5 of 7).

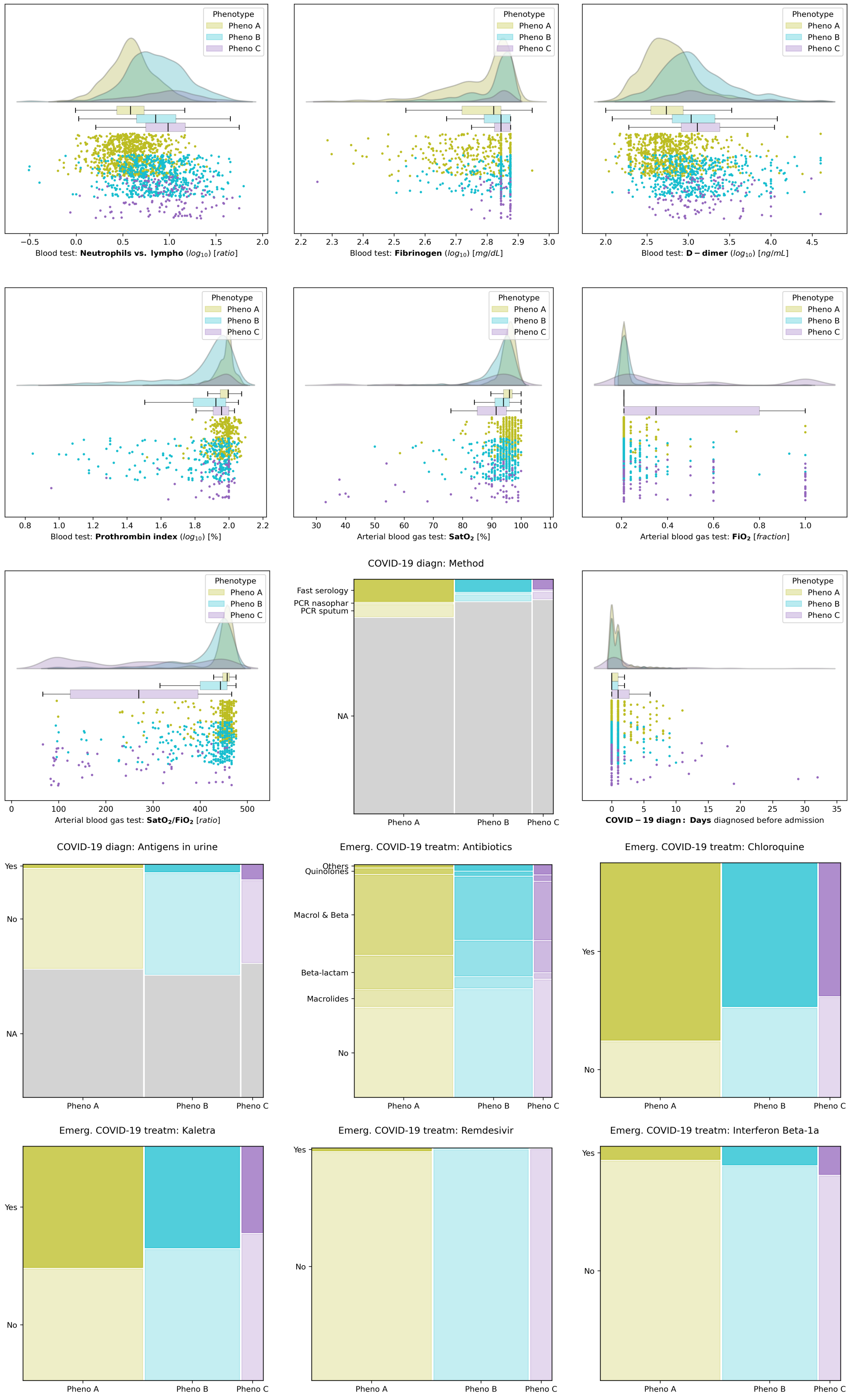

Fig. 6. Univariate descriptive graphics (6 of 7).

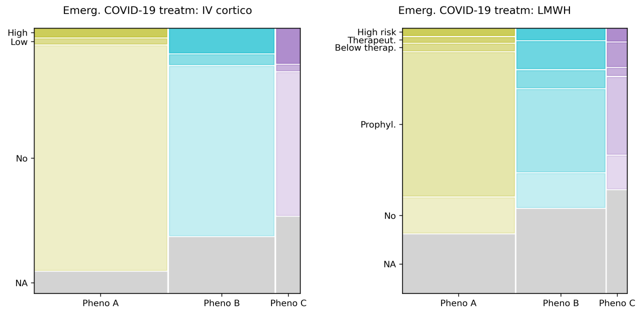

Fig. 7. Univariate descriptive graphics (7 of 7).

References

[1] W. Bergsma. A bias-correction for Cramér's V and Tschuprow's T. *J Korean Stat Soc*, 42(3):323–328, 2013.

[2] J. Cohen. *Statistical power analysis for the behavioral sciences.* Routledge, 2013.
